# Variation in suppression of black‐grass by modern and ancestral cereal root exudates

**DOI:** 10.1111/plb.70010

**Published:** 2025-03-26

**Authors:** D. T. Hickman, D. M. Withall, J. C. Caulfield, D. Comont, K. Ritz, P. Neve, A. Rasmussen, M. A. Birkett

**Affiliations:** ^1^ Swedish University of Agricultural Sciences Ultuna Uppsala Sweden; ^2^ Rothamsted Research Harpenden Hertfordshire UK; ^3^ University of Nottingham Sutton Bonington Leicestershire UK; ^4^ University of Copenhagen Taastrup Denmark

**Keywords:** Allelopathy, *Alopecurus myosuroides*, ancestral lines, benzoxazinoids, wheat

## Abstract

- This study aimed to determine the variability of hexaploid wheat (*Triticum aestivum*), ancestral diploid wheat (*T. monococcum*) and rye (*Secale cereale*) root exudate potential to inhibit the arable weed black‐grass (*Alopecurus myosuroides*), in relation to variability in resistance to herbivorous pests and pathogens across the cereal germplasm. As benzoxazinoids are suggested to play a role in resistance against these stressors, and in allelopathy, we also aimed to identify compounds in root exudates.
- We conducted *in vitro* and glasshouse bioassays to determine the efficacy of a wide range of crude cereal root exudates and their constituent compounds in inhibiting black‐grass in both axenic and biologically‐active media. LC–MS analysis was used to characterise constituents of these exudates and differences between hexaploid wheat, diploid wheat and rye.
- Root development of black‐grass was suppressed to various degrees by crude root exudates from these cereals, with the most effective being *S. cereale* var. Edmondo and *T. monococcum* MDR037. Benzoxazinoid content of root exudates varied, with ancestral wheat lines and rye exuding fewer of these compounds than hexaploid wheat, but with higher variability between lines. Co‐culture with *T. aestivum* var. Gravity significantly inhibited early shoot growth and biomass of black‐grass seedlings, but individual benzoxazinoids had no effect on black‐grass in the same system.
- These data provide evidence that cereal–black‐grass interactions are influenced by root exudates, but that their effects cannot be replicated through direct application of individual constituent compounds.

## Graphical abstract

Root exudates of commercial and ancestral cereals inhibit black‐grass and are more consistent inhibitors than application of their constituent allelochemicals.

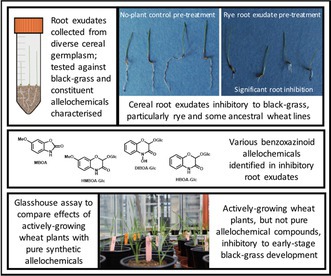

## INTRODUCTION

Cereals roots synthesise and exude many compounds, including some with inhibitory (allelopathic) potential against other organisms (allelochemicals), leading to interest in their applicability in weed management (Steinsiek *et al*. 1982; Bertholdsson 2012). Moreover, the effective developmental inhibition activity of allelochemicals like benzoxazinoids, and the cereals which synthesise and exude them, has been recognised against organisms from multiple kingdoms: plants, such as common wild oat (*Avena fatua*) and white mustard (*Sinapis alba*), as well as herbivorous pests, such as Russian wheat aphid (*Diuraphis noxia*) and pathogenic *Fusarium* species (Wouters, Gershenzon, & Vassão 2016; Hickman *et al*. 2021). Indeed, increased benzoxazinoid concentrations in modern wheat tissues have been linked to resistance to both aphids and fungal root pathogens (Gianoli & Niemeyer 1998; Wilkes *et al*. 1999). Crop breeding for increased yield has reduced the genetic variability and defence potential of modern cereal varieties compared to their ancestors (Kellner *et al*. 2010). This means that ancestral lines might produce different levels or profiles of allelochemicals, with different efficacies against target plant species. Such varying concentrations are therefore also likely to explain the common trend of higher resistance of ancestral diploid wheat genotypes to pathogens and pests compared to hexaploid wheats. For example, the diploid Einkorn wheat lines, *T. monococcum* MDR031 and MDR043 (McMillan *et al*. 2014), are resistant to the soil‐borne pathogenic fungus, Take‐all (*Gaeumannomyces graminis* var. *tritici*), while aphid growth is affected by two other lines, MDR045 and MDR049 (Elek *et al*. 2009). Other ancestral lines, like MDR037, appear to have more limited defence capability against these biotic stressors, likely related to secondary metabolite synthesis (Jing *et al*. 2007; Elek *et al*. 2009; McMillan *et al*. 2014).

It is therefore hypothesised that ancestral diploid wheat genotypes will vary in their root exudate composition, having potential value in future breeding efforts. Previous works have examined exudation of the relict wheat species, *T. spelta* (Quader *et al*. 2001), and benzoxazinoid content of some modern hexaploid wheats (Macías *et al*. 2004; Vieites‐Álvarez *et al*. 2023), but root exudates have not previously been compared across a wide range of both hexaploid and diploid cereal lines.

**Fig. 1:**
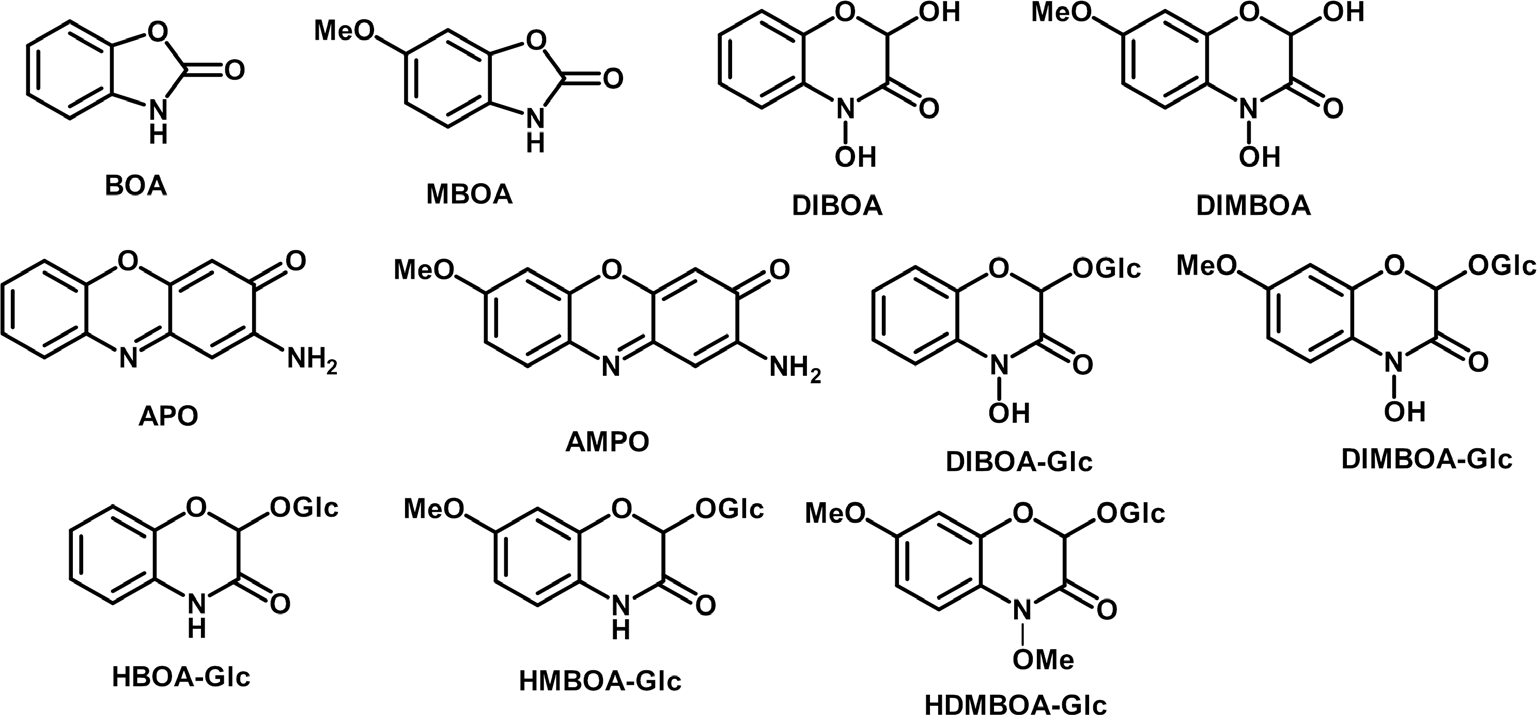
Structural formulae of cereal benzoxazinoid compounds with allelopathic potential identified and screened in this study.

The most common identified allelochemicals in cereals are benzoxazinoids, such as DIMBOA (2,4‐dihydroxy‐7‐methoxy‐1,4‐benzoxazin‐3‐one) and DIBOA (2,4‐dihydroxy‐1,4‐benzoxazin‐3‐one) (Fig. 1), although other compounds, such as phenolic acids, have also been suggested as putative allelochemicals (e.g. Wu *et al*. 2001). The allelopathic mechanism of benzoxazinoids is subject to some debate (Wouters, Gershenzon, & Vassão 2016), but may be related to the inhibition of H^+^ATPases, or other enzyme activities (Friebe *et al*. 1997; Wouters, Gershenzon, & Vassão 2016). A major determinant in allelochemical potency is delivery into, and stability within, the soil matrix as a result of microbe‐mediated rhizospheric degradation (Cipollini *et al*. 2012). In this regard, DIMBOA and DIBOA are short‐lived allelochemicals, degrading within a few days in this environment (Macías *et al*. 2004; Macías, Oliveros‐Bastidas, *et al*. 2005). Allelopathic interactions may therefore be altered, depending on allelochemical persistence in the soil, although residence time required for plant inhibition is little‐studied and probably context‐dependent. It is therefore essential to confirm allelopathic effects in biologically‐active soil, following their elucidation in axenic media (Inderjit *et al*. 2001). As microbial degradation dynamics will also be affected by the presence of other compounds, it is also essential to examine differences in allelopathic activity between crude exudates and individual allelochemicals in soil.

Black‐grass (*Alopecurus myosuroides*, Poaceae), is a major weed of cereal agriculture in Europe, resulting in large yield losses through resource competition (Naylor 1972). Management of black‐grass is challenging because of widespread evolution of resistance to multiple herbicide modes of action (Franco‐Ortega *et al*. 2021). It has been suggested that crop species' natural defence capabilities *via* allelopathy could be used to suppress black‐grass (Bertholdsson 2012), which is of particular importance as few other viable options remain for managing such herbicide‐resistant weeds (Hickman *et al*. 2023). The variability in composition and allelopathic potential of cereal root exudates for black‐grass control therefore deserves further consideration.

Hence, gaps in knowledge exist on: (i) the allelopathic potential of diverse (including ancestral) cereal germplasm towards a problematic weed like black‐grass, (ii) the persistence of these inhibitory effects in biologically active media, and (iii) differences in benzoxazinoid exudation across cereal germplasm. In consideration of these knowledge gaps, we examined the effects of hexaploid wheat (*T. aestivum*), diploid wheat ancestors (*T. monococcum*), and rye (*S. cereale*) root exudates on black‐grass development in axenic conditions, as well as their benzoxazinoid composition. We explored allelopathic responses in both herbicide‐susceptible and ‐resistant black‐grass populations, as these are hypothesised to also differ in tolerance to allelochemicals. We also investigated the allelopathic activity of both crude root exudates and synthetic benzoxazinoids on soil‐grown black‐grass, to determine the impact of soil microbial communities on allelochemical activity.

## MATERIAL AND METHODS

### Plant material

Black‐grass (*A. myosuroides* Huds.) was included in experiments as the ‘recipient’ species, but was also examined for its own root exudate profile. This material was sourced from collections at Rothamsted Research. UK: Rothamsted‐17 was used as a standard, herbicide‐susceptible population (Moss *et al*. 2004), while Peldon‐13 was included to confirm that inhibition was consistent in an herbicide‐resistant black‐grass field population (Kemp *et al*. 1990).

Hexaploid wheat (*T. aestivum* L.) was used for testing crude root exudate composition and allelopathy towards black‐grass, primarily with the commercial cultivar ‘RGT Gravity’ (Dalton Seeds, Eye, Suffolk, UK), but root exudate profiles of cultivars, ‘Costello’, ‘Firefly’, ‘Gleam’, ‘Graham’, ‘Siskin’, ‘Skyscraper’, ‘Spotlight’, ‘KWS Zyatt’ (also from Dalton Seeds) and ‘Cadenza’ (from existing stock at Rothamsted Research), were also collected for comparison (Tables S1 and S3a). Watkins landrace wheat lines 258, 546, 624, 777 and 821 (also sourced from stock at Rothamsted Research) were also screened for variation in root exudate composition, for comparison with commercial varieties (Tables S1 and S3b). This germplasm was collected prior to modern breeding, thereby capturing a higher degree of genetic diversity (Wingen *et al*. 2014), and we hypothesised there would be increased variation in root exudate profiles compared with modern elite varieties.

Diploid wheat (*T. monococcum* L.) was sourced from existing collections at Rothamsted Research (but see Jing *et al*. 2007 for details of origin). MDR037 (*T. monococcum* var. *macedonicum*, spring wheat, collected from Armenia, 1934), MDR043 (*T. monococcum* var. *monococcum*, spring wheat, collected from Greece, 1950) and MDR049 (*T. monococcum* var. *pseudohornemannii*, winter wheat, collected from Iran, year unknown), were tested for crude root exudate allelopathy towards black‐grass. Further, these and the additional lines MDR031 (*T. monococcum* var. *monococcum* x *macedonicum*, spring wheat collected from Turkey, 1931) and MDR045 (*T. monococcum* var. *vulgare*, spring wheat collected from Denmark, 1970) were screened for root exudate composition (Table S1). These lines were of interest, given their differences in defence capability against non‐plant biotic stressors, as previously described.

Rye (*S. cereale* L.) was included in examination of allelopathic potential and root exudate profile as a comparison to the wheat species described above, and specifically the modern commercial cultivar, ‘Edmondo’ (Dalton Seeds). Rye is thought to exude high levels of benzoxazinoids (Burgos & Talbert 2000), and therefore served as a positive control.

### Seedling pre‐treatment

All plant seed material used throughout this investigation was surface‐sterilised using a protocol derived from Speakman & Krüger (1983), where seeds were immersed in 10 ppm oxytetracycline hydrochloride (Thermo Fisher Scientific, Loughborough, UK) for 20 h, then 0.1% silver nitrate (Avocado Research Chemicals, Altrincham, UK) for 10 min, before being rinsed with 0.5% sodium chloride (Thermo Fisher), then triple‐rinsed with autoclaved distilled water. Seeds were then pre‐germinated on Parafilm‐sealed 0.8% water agar plates in a light/dark regime of 17 °C/11 °C for 14/10 h in a Sanyo MLR‐351 versatile environment chamber. Black‐grass seeds were pre‐germinated for 5 days, while all cereal seeds were pre‐germinated overnight because they develop faster. This ensured that all seedlings were sown into experimental assays at the point of radicle emergence.

### Soils

Soil used in this investigation comprised:

1. A Kettering loam (hereafter described as ‘Weed mix’), used as standard field soil for glasshouse trials of black‐grass at Rothamsted Research (Comont *et al*. 2019). This Weed mix was sieved through a 1‐mm sieve before use.
2. Soils collected from the Highfield Long‐term Experiment at Rothamsted Research (UK grid reference 51.804393, −0.362667), specifically from three different land uses: bare fallow, grassland, and wheat field. The soil at this site is silty clay loam (25% clay: 62% silt: 13% sand; Chromic Luvisol according to FAO criteria). These plots were established in 1959, after which a consistent and representative microbial community developed for each land use (Hirsch *et al*. 2017). Approximately 150 g soil was taken from each plot, (three plots for each of the three land uses; Figure S1), partially oven‐dried to increase friability, and sieved through a 1 mm sieve before use.

### *In vitro* inhibition of black‐grass within sterilised sand and soil

**Fig. 2:**
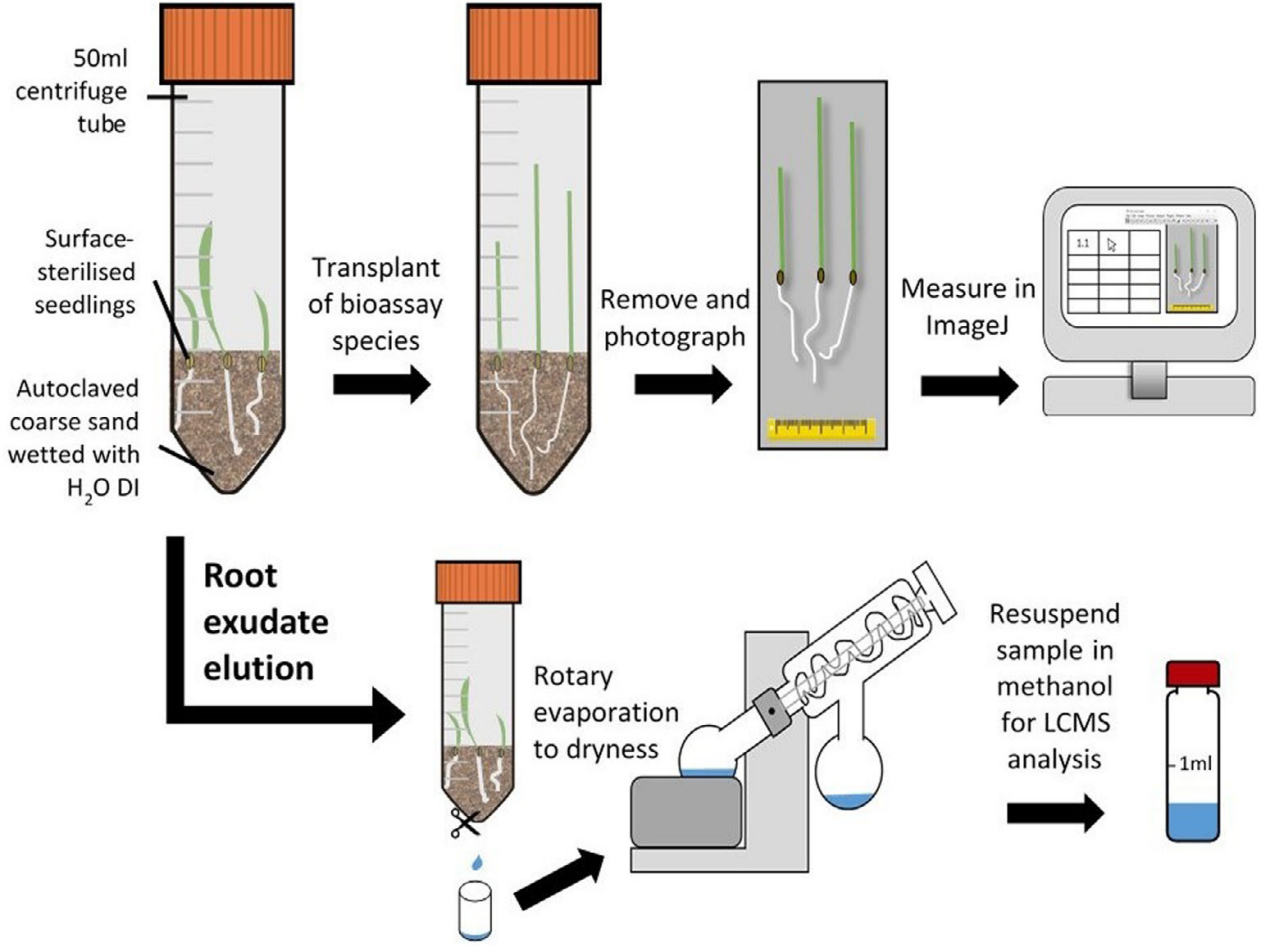
Schematic of experiments involving the sand centrifuge tube system, both in terms of bioassays of crude exudates (upper row) and characterisation of their chemical composition (lower row).

The response of black‐grass seedlings to root exudates from the chosen modern hexaploid wheat, diploid wheat and rye was assessed using sterile Falcon® centrifuge tubes (50 mL) filled with 15 g autoclaved coarse sand (Aggregate Industries UK, Coalville, UK) moistened with autoclaved de‐ionised water (5 ml; Purite Veolia, Thame, UK) (Fig. 2). This assay followed methods used elsewhere to profile metabolites in rhizosphere soil (see Petriacq *et al*. 2017). All assays were incubated in a light/dark regime of 17 °C/11 °C for 14/10 h in stable humidity in the versatile environment chamber. For each experiment, ‘donor’ plants (either pre‐germinated hexaploid wheat, diploid wheat or rye seedlings, 4 seedlings per tube) were sown into the sand, tubes sealed, and then incubated for 7 days to maximise allelochemical concentration (according to Zúñiga & Massardo 1991). No‐plant controls comprised tubes containing only moistened sand incubated for 7 days. After this, donor plants were removed and replaced with recipient pre‐germinated black‐grass seedlings (4 plants per tube), grown for a further 7 days. All recipient plants were photographed at the end of the growth period, using a photography stage and standardised lighting to maintain a consistent scale. Root and shoot lengths were then measured using ImageJ through the FIJI open‐source project (v. 1.53c; Schindelin *et al*. 2012), and resulting data analysed in R (v. 4.4.2; R Core Team 2024). Root and shoot lengths were modelled using linear mixed effect models (‘lme4’ package), with replicate as a random factor and different donor species used in pre‐treatment and, in some cases the different recipient black‐grass populations, as fixed factors. Analyses specifically tested the root and shoot length of black‐grass, and wheat where relevant. *P*‐values were derived from Satterthwaite approximations using the package ‘lmerTest’. Tukey's post‐hoc analyses were then conducted using ‘emmeans’, with significant post‐hoc comparisons denoted on figures as letters, derived from the package ‘multcompView’, and depicted graphically in bar charts using ‘ggplot2’ in R for visual interpretation. In further experiments, the bioassay setup was adapted to contain biologically active field soil, collected and prepared as described above. The following experiments were carried out (summarised in Table S2):

Experiment 1: To assay hexaploid wheat and rye for allelopathic potential, *T. aestivum* var. Gravity and *S. cereale* var. Edmondo were sown into Falcon tubes containing 15 g sterilised sand and grown for 7 days. A no‐plant control treatment was also included. These pre‐treatments were followed by pre‐germinated recipient seedlings of either black‐grass Rothamsted‐17 or Peldon‐13 for the same length of time.

Experiment 2: To compare diploid wheat lines with rye and hexaploid wheat in terms of allelopathic potential, *T. aestivum* var. Gravity and *S. cereale* var. Edmondo, as well as *T. monococcum* MDR lines 037, 043 and 049, were sown into Falcon tubes containing 15 g sterilised sand and grown for 7 days. A no‐plant control was also included. These pre‐treatments were followed by pre‐germinated recipient seedlings of Rothamsted‐17 black‐grass for the same length of time.

Experiment 3: To assay hexaploid wheat, diploid wheat and rye for allelopathic potential in soil, *T. aestivum* var. Gravity, *S. cereale* var. Edmondo, *T. monococcum* MDR037 and black‐grass Rothamsted‐17 were sown into Falcon tubes containing 20 g Weed mix soil and grown for 7 days. A no‐plant control was also included. These pre‐treatments were followed by pre‐germinated recipient seedlings of Rothamsted‐17 black‐grass.

Prior to this assay, water‐holding capacity (WHC) was calculated for the Weed mix soil. This soil was then wetted to 25% WHC, and tubes incubated for 2 weeks under conditions consistent with pre‐germination (light/dark regime 17 °C/11 °C, 14/10 h light) to facilitate development of a stable microbial community (Xue *et al*. 2017). The plant bioassay was initiated by increasing WHC to 50%, a value derived from WHC measurements of the Highfield experiment (M. Khandoker, unpublished data).

Experiment 4: To assay the allelopathic potential of synthetic benzoxazinoids in soil, DIBOA and DIMBOA were applied at discriminatory doses to herbicide‐susceptible black‐grass Rothamsted‐17. These concentrations of DIMBOA and DIBOA were derived from dose–response analyses on black‐grass Rothamsted‐17 (Hickman *et al*. unpublished data), which in both cases (allowing for dilution effects of pre‐wetted soil) had previously been found to be sufficient to produce consistently significant detrimental effects on root growth. These chemicals were applied as solutions in DMSO (0.5%) (Thermo Fisher) at 500 μM for DIBOA and 1000 μM for DIMBOA. These DMSO concentrations were sufficient to effectively dissolve both compounds, without being too concentrated to significantly affect plant development when added to the pre‐wetted soil (Schmitz & Skoog 1970). DMSO (0.5%) alone was used as control. Methods used for synthesis of DIMBOA and DIBOA can be found in Appendix S3. These compounds were selected given their well‐substantiated allelopathic potential (Quader *et al*. 2001; Zhang *et al*. 2016), including specifically towards black‐grass (Yang *et al*. 2020), and their common presence in exudates collected from wheat and rye roots (e.g. Mwendwa *et al*. 2021; Hazrati *et al*. 2022). The seedlings were grown in Weed mix, Highfield bare fallow, Highfield grassland or Highfield wheat arable soil. Soil was pre‐treated as described above, and 20 g soil was added to each centrifuge tube, except for those containing the less dense grassland soil, where only 15 g were added to allow sufficient aboveground growth space for plants.

### Root exudate collection

To collect root exudates for LC–MS analysis, a simple assay with modifications to the previously described bioassay system (*sensu* Petriacq *et al*. 2017) was used. Centrifuge tube bases were pierced using a sterilised scissor blade (99% acetone; Thermo Fisher), and eluted rapidly (within 1 min, to minimise damage to roots), using HPLC grade methanol (Thermo Fisher), then diluted to 70% using deionised water. Eluates were evaporated to dryness *in vacuo* using a Büchi R‐300 Rotavapor rotary evaporator then resuspended in HPLC grade methanol (0.5 mL) prior to analysis.

### LC–MS analysis of root exudates

The LC–MS analysis was conducted with an Acquity ultra‐high‐pressure liquid chromatography (UPLC) system coupled to a Synapt G2Si Q‐Tof mass spectrometer with an electrospray ionisation source (Waters, Wilmslow, UK). Masslynx 4.1 software (Waters) was used to control the system. Chromatographic separation was performed at a flow rate of 0.21 mL min^−1^ using a UPLC BEH C18 column (2.1 × 150 mm, 1.7 μm; Waters), coupled to a C18 Vanguard pre‐column (2.1 × 5 mm, 1.7 μm; Waters). The mobile phase consisted of solvent A (0.02% formic acid v/v, water) and solvent B (0.02% formic acid v/v in methanol), with the following gradient: initial conditions 95% A, 0–3 min 95% A, 3–7 min 85% A, 7–11 min 75% A, 11–13 min 70% A, 13–15 min 70% A, 15–18 min 50% A, 18–21 min 50% A, 21–25 min 25% A, 25–30 min 0% A, 30–39 min 0% A, 39–39.1 min 95% A, 39.1–43 min 95% A. The column was maintained at 50 °C and the injection volume was 1 μl. Samples were analysed in both positive and negative ion modes with two consecutive injections of methanol between modes for stabilisation. An Acquity photodiode array (PDA) detector monitored the UV trace (range 200–450 nm), sampling rate of 10 points s^−1^, with resolution set to 2.4 nm. The LC–MS system was set to a mass range of 50–1200 Da and scan time of 0.1 s in both ionisation modes. The LC–MS instrument was operated with the following conditions: capillary voltage –2.5KV, sample cone voltage 30 V, sample offset 80 V, source temperature 100 °C, desolvation temperature 300 °C, desolvation gas flow 800 L h^−1^, cone gas flow 57 L h^−1^. Compound identifications were confirmed using standards, where these could be successfully synthesised (DIMBOA, DIBOA, MBOA, BOA, APO, AMPO; see Appendix S3). Where synthesis was not possible, tentative identifications were made using existing literature (specifically Adhikari *et al*. 2012; Bruijn *et al*. 2016; Hama *et al*. 2022; Hanhineva *et al*. 2011; Hazrati *et al*. 2021; Macías, Oliveros‐Bastidas, *et al*. 2005; Wouters, Blanchette, *et al*. 2016).

### Inhibition of black‐grass in soil under glasshouse conditions

**Fig. 3:**
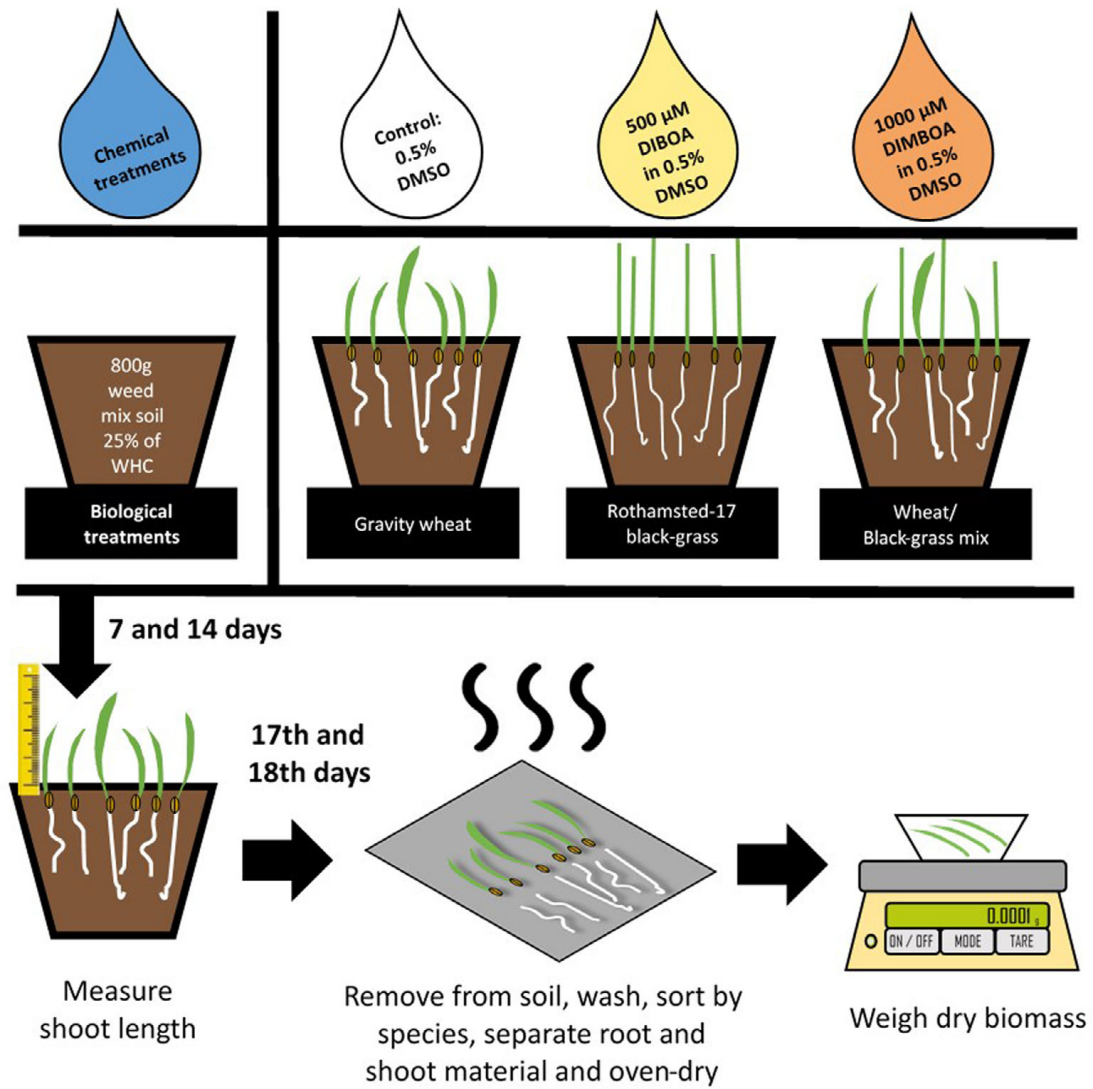
Schematic of glasshouse assay experimental setup, processing and data collection. All combinations of biological and chemical treatments were tested, with six replicates of each (n = 54), and blocked by replicate during the growth period to minimise location effects within the compartment.

A glasshouse experiment was established to confirm results from experiments 1 to 4 in a larger pot‐based assay (Fig. 3). This was again conducted under controlled day/night cycles of 17 °C/11 °C for 14/10 h. Pots (15 cm diameter) were filled with Weed mix soil (800 g), sown with three biological treatments, specifically *T. aestivum* var. Gravity, black‐grass Rothamsted‐17, and a mixed treatment of these two biotypes. All plants were established in compost seed trays, for 9 days for black‐grass and 5 days for *T. aestivum*, prior to transplantation into the pots of Weed mix soil. Pots were thoroughly wetted 1 day prior to plant sowing. Six seedlings were grown on in each biological treatment, including three of each species in the mixed treatment. These treatments were left for 1 day to allow plant establishment before one of three chemical treatments was applied. These treatments were 1000 μM DIMBOA in 0.5% DMSO, 500 μM DIBOA in 0.5% DMSO, or a control of 0.5% DMSO, as previously described in Experiment 4. Treatments were applied in a volume of liquid consistent with increasing the water content of each pot from 25% WHC to 50% WHC. Pot placement was randomised on a bench in a glasshouse, with positions blocked by replicate. All combinations of biological and chemical treatment were included in each replicate, and six replicates of each treatment combination were established (3 biological treatments ×3 chemical treatments ×6 replicates, n = 54). All pots were watered uniformly with tap water to maintain soil moisture.

Plant heights were measured at 7 and 14 days after treatment (DAT) to capture temporal variations in allelopathic effects and developmental responses (Weidenhamer *et al*. 2014). At 17–18 DAT, plants were removed for root washing, drying, and biomass measurement. Root and shoot material were separated, dried overnight at 80 °C, and total dry weight of all roots and shoots of each species per pot recorded. The sum of these values was also used for the metric ‘total biomass’. Growth of both black‐grass and wheat root and shoot biomass under different chemical treatments was then analysed by ANOVA and compared between biological treatments, as previously described.

## RESULTS

### Axenic tube sand bioassays with root exudates

**Fig. 4:**
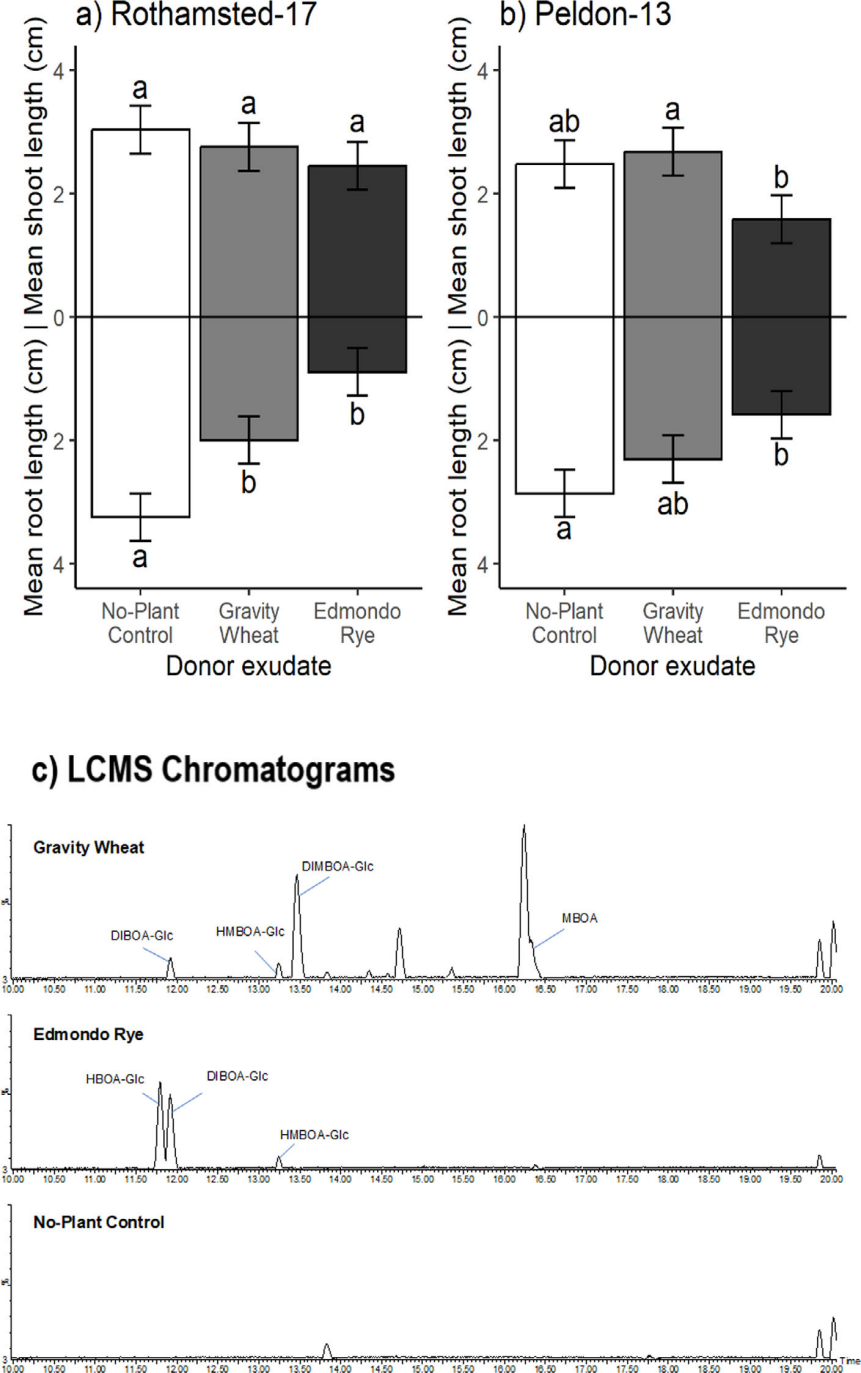
Upper: Mean 7‐day shoot and root lengths (cm) of black‐grass, *Alopecurus myosuroides* Rothamsted‐17 (herbicide‐susceptible) (a), and Peldon‐13 (herbicide‐resistant) (b) in axenic sand system, in response to root exudates from common wheat, *Triticum aestivum* var. Gravity, and rye, *Secale cereale* var. Edmondo (n = 8). Wheat and rye seedlings were grown in the sand system for 7 days then removed prior to addition of pre‐germinated black‐grass seedlings. Error bars ± SEM, letters indicate significant differences. (c) Total ion chromatograms from LC–MS analysis of collected root exudates of Gravity wheat and Edmondo rye, with tentatively identified benzoxazinoids labelled.

Experiment 1: For plants assayed in controlled environment conditions in sterilised sand, application of growth media containing root exudates from common wheat, *T. aestivum* var. Gravity, and rye, *S. cereale* var. Edmondo, to herbicide‐susceptible Rothamsted‐17 and herbicide‐resistant Peldon‐13 black‐grass, resulted in no significant differences in mean shoot length between treatments and the no‐plant control ‘exudate’ (Fig. 4a,b). Rye root exudates were significantly inhibitory to Peldon‐13 shoots compared to wheat root exudates, however, and the two black‐grass populations were also significantly different in shoot length, independent of root exudate treatment. Root length was significantly affected by root exudate treatment in both black‐grass populations (Table 1). Specifically, both Rothamsted‐17 and Peldon‐13 mean root lengths were significantly inhibited by *S. cereale* root exudates (by 72.5% in Rothamsted‐17, 44.6% in Peldon‐13), while Rothamsted‐17 mean root length was also significantly reduced when treated with *T. aestivum* root exudate (by 38.5%), compared to the no‐plant control. There was no significant difference in mean root length when Peldon‐13 was treated with *T. aestivum* root exudate.

**Table 1:** Statistical output from ANOVA of mean Rothamsted‐17 and Peldon‐13 black‐grass shoot and root lengths in axenic coarse sand following pre‐treatment with different ‘donor’ cereal root exudates (no‐plant control, *Triticum aestivum* var. Gravity, or *Secale cereale* var. Edmondo).

		sum sq	mean sq	No. *df*	denom *df*	*f*‐value	*P*‐value
shoot length	‘Donor’ exudate	6.066	3.033	2	42	5.422	0.008**
	Black‐grass population	3.416	3.416	1	42	6.107	0.018*
	‘Donor’ exudate × Black‐grass population	1.137	0.568	2	42	1.016	0.371
root length	Treatment	26.126	13.063	2	35	22.368	>0.001***
	Black‐grass Population	0.551	0.551	1	35	0.944	0.338
	‘Donor’ Exudate × Black‐grass Population	2.478	1.239	2	35	2.121	0.135

*P < 0.05, **P < 0.01, ***P < 0.001.

**Fig. 5:**
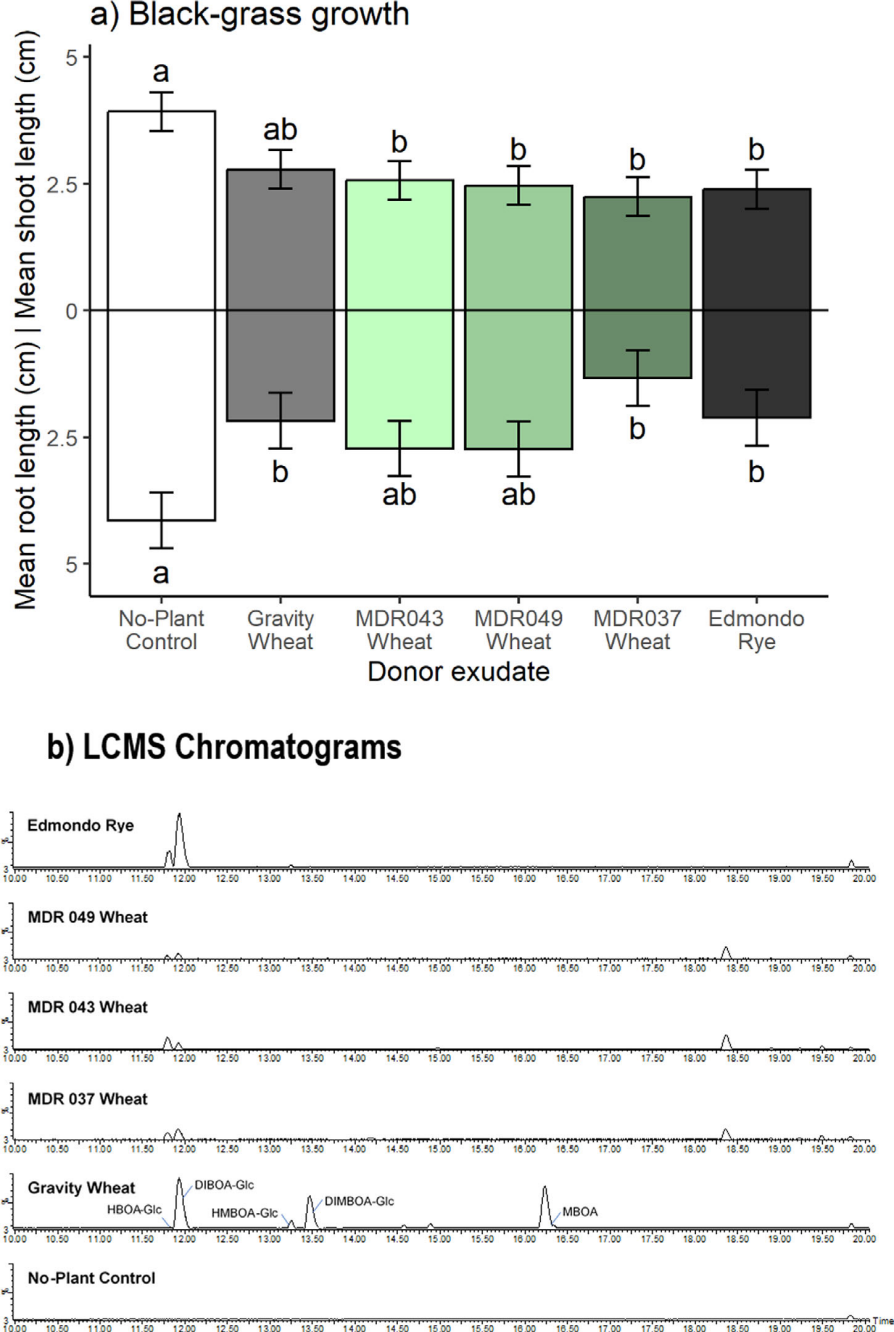
(a) Mean 7‐day shoot and root lengths (cm) of black‐grass Rothamsted‐17 (herbicide‐susceptible) in an axenic sand system in response to root exudates from hexaploid wheat (*Triticum aestivum* var. Gravity), diploid wheat lines (*T. monococcum* MDR043, MDR049 and MDR037) or rye (*Secale cereale* var. Edmondo). Hexaploid wheat, diploid wheat and rye seedlings were grown in the sand system for 7 days and removed prior to addition of pre‐germinated black‐grass seedlings. Error bars ± SEM, letters indicates significant differences. (b) Total ion chromatograms from LC–MS analysis of collected root exudates showing tentative identification of benzoxazinoids.

Experiment 2: In further axenic bioassays using MDR lines of diploid wheat, donor exudates significantly inhibited root growth in Rothamsted‐17 black‐grass, with post‐hoc comparisons indicating that exudates from *T. monococcum* MDR037, *T. aestivum* var. Gravity and *S. cereale* var. Edmondo were significantly different from the control treatment. All non‐control exudates, except *T. aestivum* var. Gravity, were also significantly inhibitory to black‐grass shoot growth in this assay (Fig. 5a and Table 2).

**Table 2:** Statistical output from ANOVA of mean black‐grass shoot and root lengths in axenic coarse sand following pre‐treatment with different diploid and hexaploid cereal root exudates (no‐plant control, *Triticum aestivum* var. Gravity, *T. monococcum* lines MDR037, MDR043, and MDR049, or *Secale cereale* var. Edmondo).

		sum sq	mean sq	No. *df*	denom *df*	*f*‐value	*P*‐value
shoot length	‘Donor’ Exudate	14.939	2.988	5	34.079	5.134	0.001*
root length	‘Donor’ Exudate	37.125	7.425	5	41	6.2316	>0.001***

*P < 0.05, ***P < 0.001.

### Root exudate composition

The LC–MS analysis of *T. aestivum* var. Gravity, *S. cereale* var. Edmondo and *T. monococcum* MDR037 root exudates used in axenic sand bioassays tentatively identified several benzoxazinoids (Figs 4c and 5b). For *T. aestivum*, HBOA‐Glc (2‐hydroxy‐1,4‐benzoxazin‐3‐one‐Glucoside), DIBOA‐Glc (DIBOA‐Glucoside), HMBOA‐Glc (2‐hydroxy‐7‐methoxy‐1,4‐benzoxazin‐3‐one‐Glucoside), DIMBOA‐Glc (DIMBOA‐Glucoside) and MBOA (6‐methoxy‐benzoxazolin‐2‐one), were detected (structures provided in Fig. 1); while for *S. cereale*, only HBOA‐Glc, DIBOA‐Glc and HMBOA‐Glc were detected (Fig. 4c). For *T. monococcum* lines MDR037, MDR043 and MDR049, only HBOA‐Glc and DIBOA‐Glc were detected (Fig. 5b). There were no detectable benzoxazinoids in black‐grass exudates or in no‐plant controls, as expected.

### Soil bioassays with root exudates

**Fig. 6:**
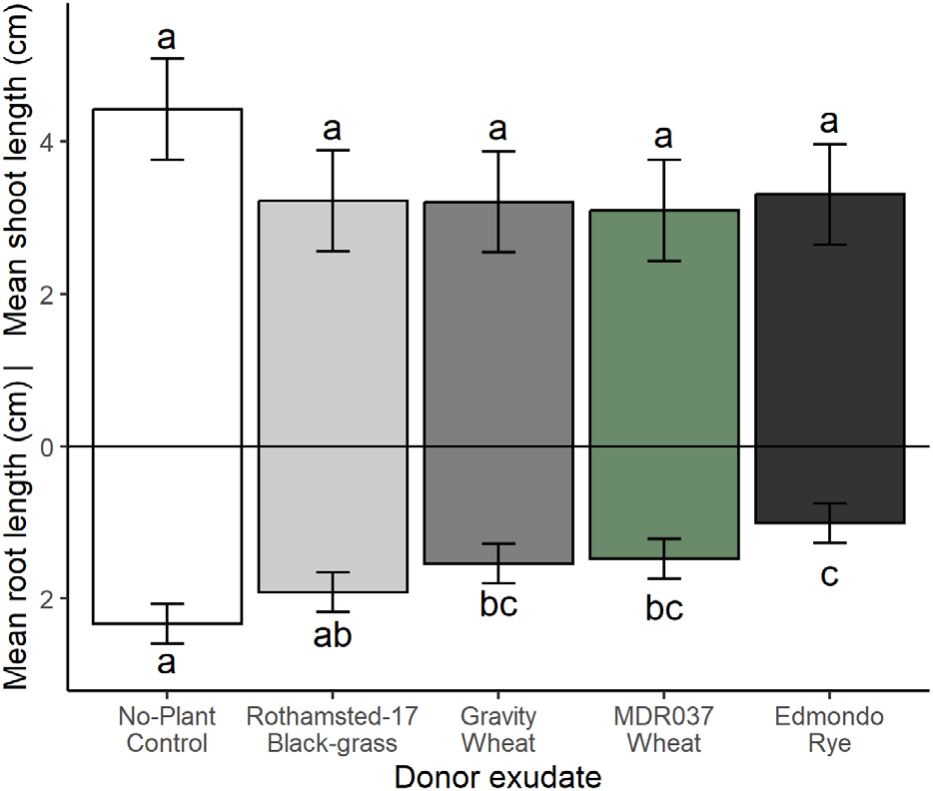
Mean 7‐day shoot and root lengths (cm) of black‐grass, *Alopecurus myosuroides* Rothamsted‐17 (herbicide‐susceptible) in a soil system containing Weed mix soil in response to root exudates from common wheat (*Triticum aestivum* var. Gravity), diploid wheat line (*T. monococcum* MDR037), or rye (*Secale cereale* var. Edmondo). Error bars ± SEM, letters indicate significant differences.

Experiment 3: For plants grown under controlled environment conditions in Weed mix soil, application of growth media containing root exudates from *T. aestivum* var. Gravity, *T. monococcum* MDR037, *S. cereale* var. Edmondo and Rothamsted‐17 black‐grass resulted in no significant difference in mean shoot length between treatments and the no‐plant control exudate (Fig. 6 and Table 3). Mean root length of black‐grass was significantly inhibited by all non‐control root exudate treatments, except for black‐grass, as reported in sand (Fig. 5).

**Table 3:** Statistical outputs from ANOVA of mean black‐grass shoot and root lengths in weed mix soil following pre‐treatment with different root exudates (no‐plant control, black‐grass, *Triticum aestivum* var. Gravity, *T. monococcum* line MDR037, or *Secale cereale* var. Edmondo).

		sum Sq	mean Sq	No. *df*	denom *df*	*f*‐value	*P*‐value
shoot length	‘Donor’ Exudate	13.888	3.472	4	28	1.977	0.126
root length	‘Donor’ Exudate	8.282	2.070	4	35	8.965	>0.001***

***P < 0.001.

### Benzoxazinoid activity in soil

**Fig. 7:**
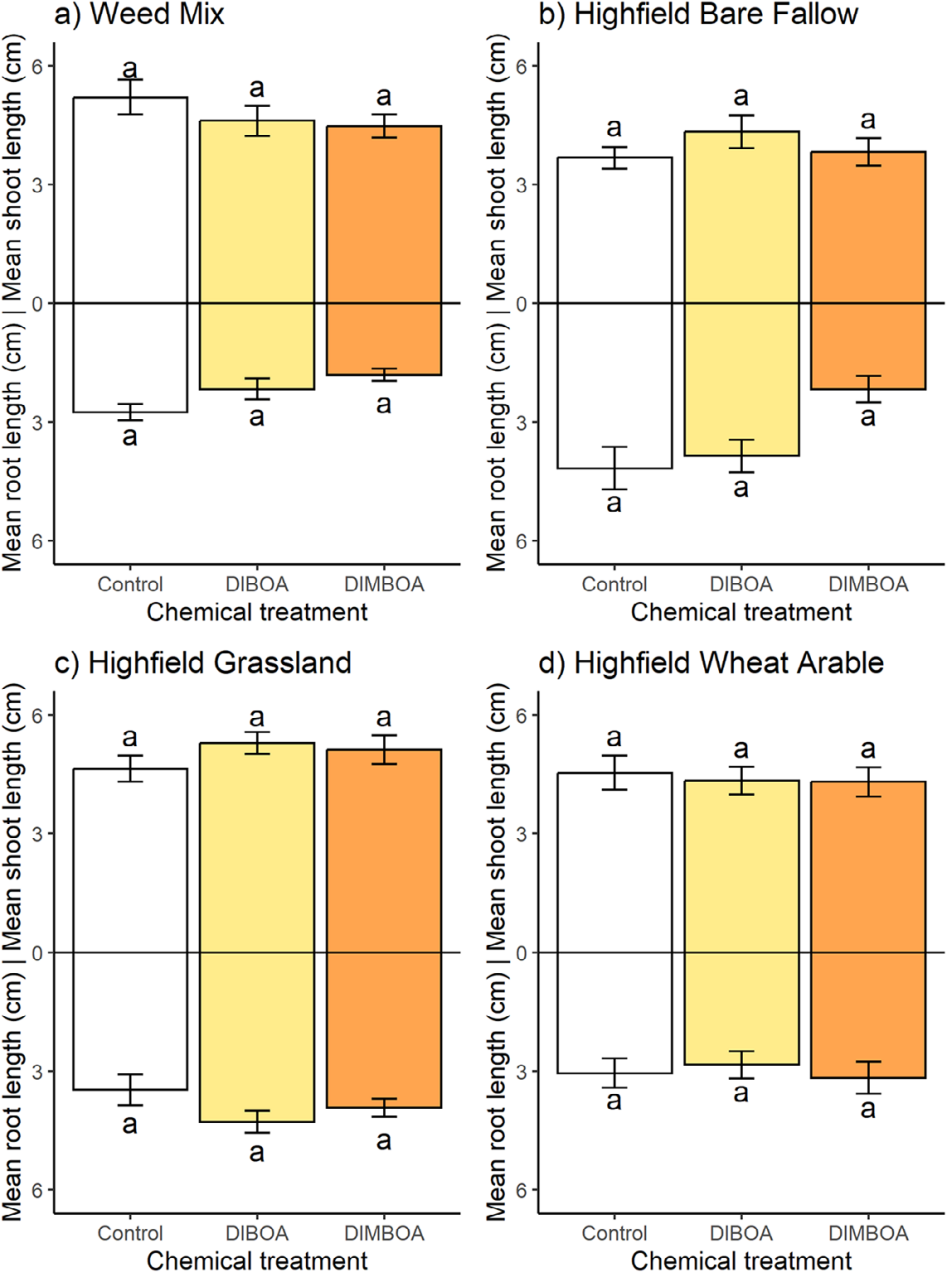
Mean 7‐day root and shoot lengths (cm) of black‐grass, *Alopecurus myosuroides* Rothamsted‐17 (herbicide‐susceptible) in an axenic soil system with differing benzoxazinoid treatments (Control = 0.25% DMSO, DIBOA = 500 μM DIBOA in 0.25% DMSO, DIMBOA = 1000 μM DIMBOA in 0.25% DMSO). (a) Weed mix control; (b) Highfield bare fallow; (c) Highfield grassland; (d) Highfield wheat arable soils. Error bars ± SEM. Letters indicates no significant differences between treatments.

Experiment 4: Soil treatment had a significant effect on both black‐grass root and shoot lengths (Table 4). However, application of DIMBOA and DIBOA at their respective discriminatory doses to herbicide‐susceptible Rothamsted‐17 black‐grass in Weed mix, Highfield bare fallow, Highfield grassland and Highfield wheat arable soil resulted in no significant difference in mean shoot and root lengths between benzoxazinoid treatments and the no‐benzoxazinoid control (Fig. 7). There was some evidence of differences in degradation rate between the different compounds and the soils tested (Appendix S2), as peaks of DIMBOA and DIBOA diminished from samples from these different growth media at varying rates.

**Table 4:** Statistical output from ANOVA of mean black‐grass shoot and root lengths after growth in different soil treatments (weed mix control, Highfield bare fallow, Highfield grassland, and Highfield wheat arable), and chemical treatments (no‐benzoxazinoid control, DIBOA or DIMBOA solution).

		sum sq	mean sq	No. *df*	denom *df*	*f*‐value	*P*‐value
shoot length	Soil treatment	8.924	2.975	3	44	2.934	0.044*
	Chemical treatment	0.430	0.215	2	44	0.212	0.810
	Soil × chemical treatment	3.920	0.653	6	44	0.645	0.694
root length	Soil treatment	21.578	7.193	3	48	8.514	>0.001***
	Chemical treatment	4.070	2.035	2	48	2.409	0.101
	Soil × chemical treatment	11.193	1.865	6	48	2.208	0.058

*P < 0.05, ***P < 0.001.

### Glasshouse assay

**Fig. 8:**
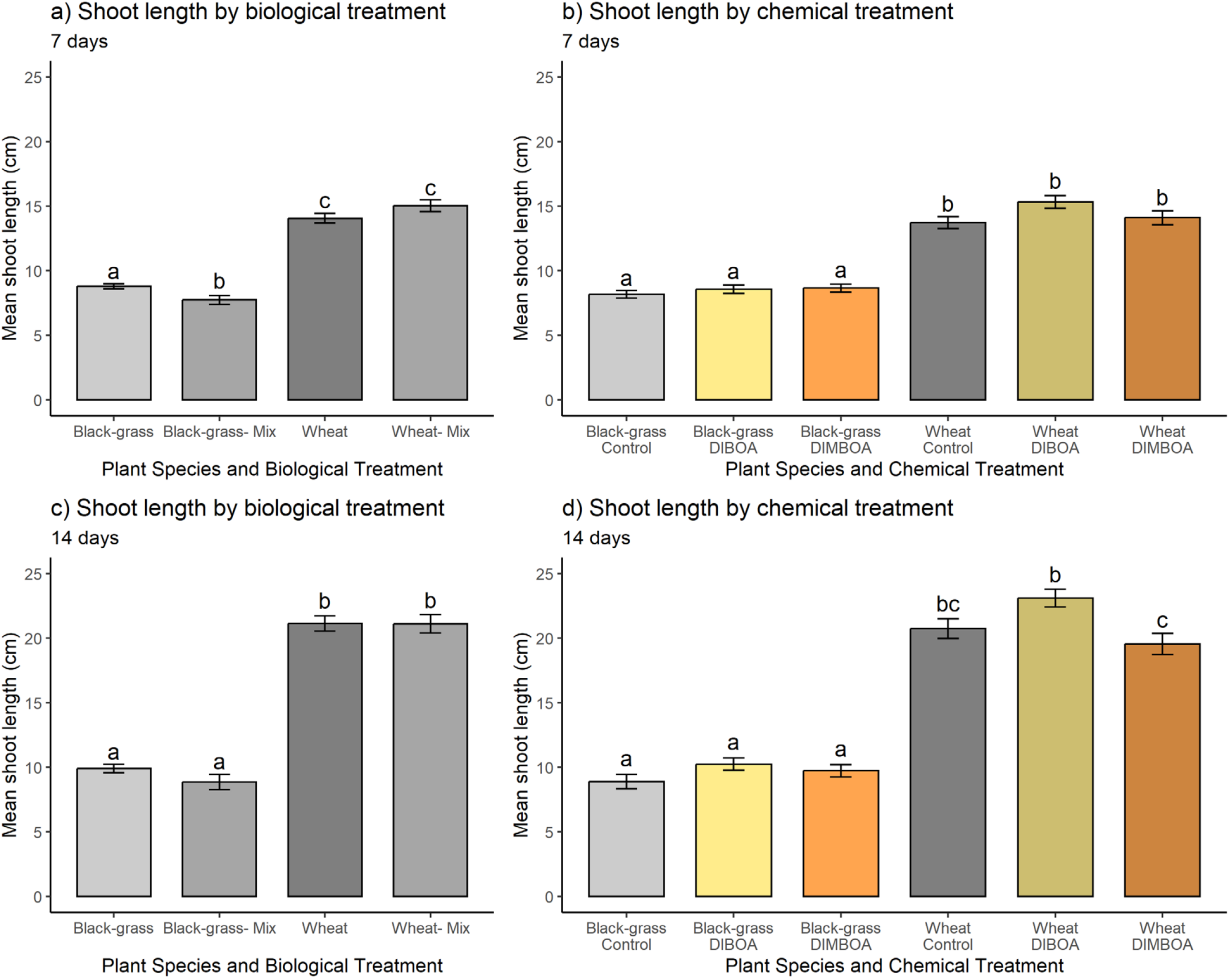
Mean 7‐ and 14‐day shoot lengths (cm) of black‐grass and wheat under (a) biological (only wheat and black‐grass or co‐culture of both species; ‘mix’, with measurements for both species compared with growth in isolation); (b) benzoxazinoid chemical treatments (control of 0.5% DMSO, 500 μM of DIBOA in 0.5% DMSO, or 1000 μM of DIMBOA in 0.5% DMSO, added to wetted soil) at 7 DAT (c), and 14 DAT (d) under glasshouse conditions. Axis labels indicate species measured followed by treatment. Pot replicates = 6 (n = 54), Individual seedlings per pot = 6 (or 3 + 3 where mixed). Error bars ± SEM, letters indicate significant differences.

**Fig. 9:**
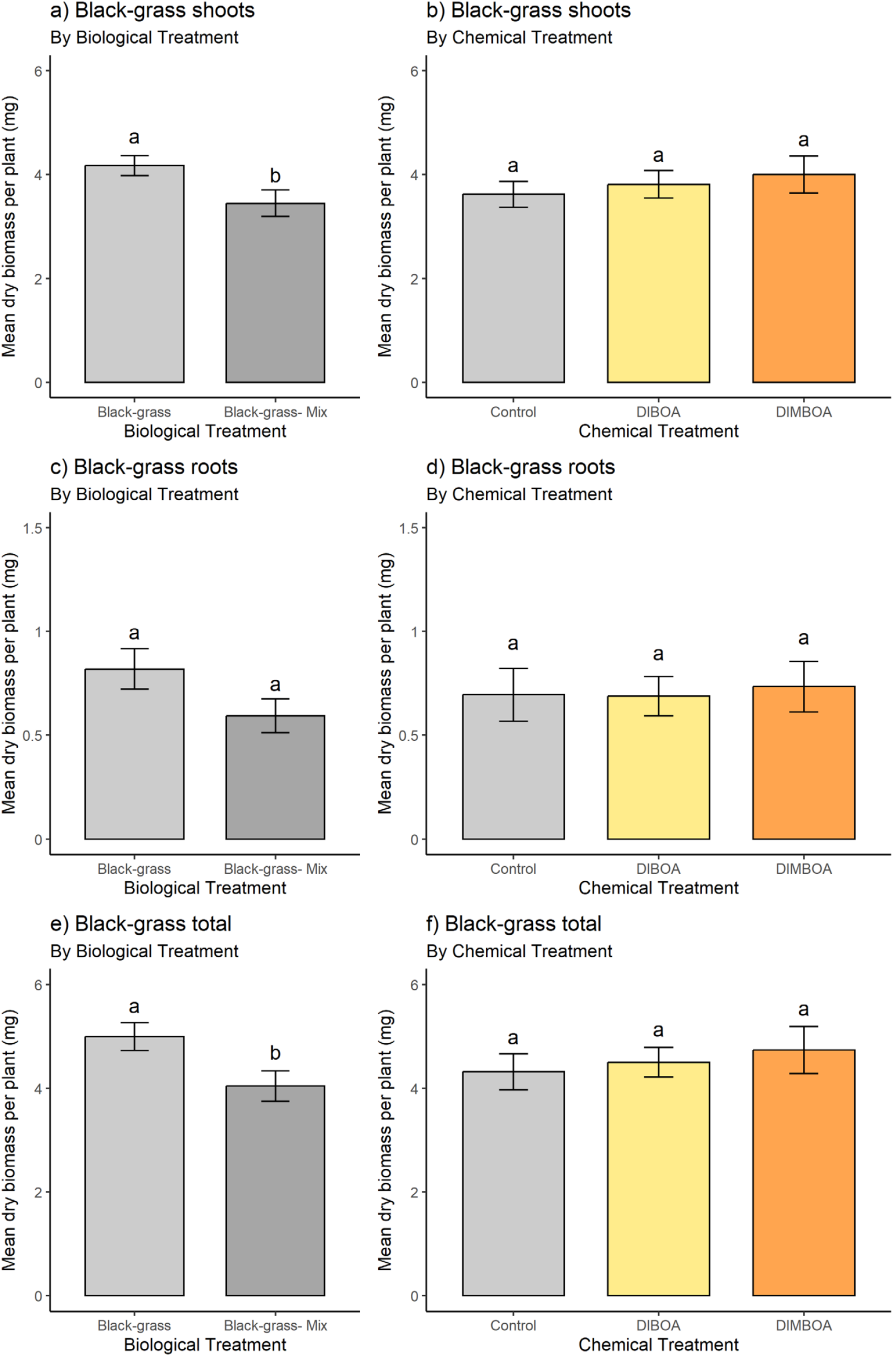
Mean dry biomass of black‐grass after 3 weeks of growth under glasshouse conditions and various treatments: (a) total biomass, (b) shoot biomass, (c) root biomass by biological treatment; (d) total biomass, (e) shoot biomass, (f) root biomass after benzoxazinoid chemical treatment. Pot replicates = 6 (n = 54), Individual seedlings per pot = 6 (or 3 + 3 where mixed). Error bars ± SEM, letters indicate significant differences from corresponding controls. Corresponding graphs for wheat grown in the same experiment can be found in Supplementary material S2.3.

Shoot length at 7 and 14 DAT: the presence of wheat in co‐culture with black‐grass (‘mix’ treatment) had a detrimental effect on black‐grass shoot length at 7 DAT, compared with the control treatment where wheat was not present (Fig. 8a and Table 5). Conversely, black‐grass was not detrimental to wheat shoot growth (comparing ‘mix’ to ‘control’ for wheat). Neither black‐grass nor wheat shoots were significantly affected by chemical treatment, in coherence with previous results in soil (Fig. 9b). Wheat was also unaffected by black‐grass at 14 DAT (Fig. 8c), while the previously significant inhibition of black‐grass shoot length by wheat became insignificant at this later time point. Again, neither benzoxazinoid chemical treatment was significantly inhibitory towards black‐grass or wheat compared to controls at 14 DAT (Fig. 8d).

**Table 5:** Statistical outputs from ANOVA of mean black‐grass and wheat shoot lengths after 7 and 14 days of growth under glasshouse conditions for both biological treatments (homogeneous black‐grass or wheat, or a mix of the two), and chemical treatments (no‐benzoxazinoid control, DIBOA or DIMBOA solution).

			sum sq	mean sq	No. *df*	denom *df*	*f*‐value	*P*‐value
black‐grass	Day 7	Biological treatment	13.264	13.264	1	29	7.571	0.010*
		Chemical treatment	1.019	0.509	2	29	0.291	0.750
		Biological × chemical treatment	4.538	2.269	2	29	1.295	0.289
	Day 14	Biological treatment	7.200	7.200	1	27	1.766	0.195
		Chemical treatment	6.231	3.115	2	27	0.764	0.476
		Biological × chemical treatment	4.294	2.147	2	27	0.527	0.597
wheat	Day 7	Biological treatment	8.298	8.298	1	25	1.525	0.228
		Chemical treatment	17.656	8.828	2	25	1.622	0.218
		Biological × chemical treatment	6.462	3.231	2	25	0.594	0.560
	Day 14	Biological treatment	0.029	0.029	1	30	0.003	0.960
		Chemical treatment	74.547	37.273	2	30	3.650	0.038*
		Biological × chemical treatment	15.015	7.507	2	30	0.735	0.488

*P < 0.05.

Dry biomass at termination: dry weights of plants measured after 3 weeks of growth were coherent with shoot length measurements at 7 DAT. The wheat–black‐grass co‐culture (‘mix’) culminated in significantly inhibited total black‐grass biomass (Fig. 9a and Table 6). Neither the constituent root nor shoot biomass was significantly reduced in this treatment, however (Fig. 9b, c). Benzoxazinoid chemical treatments did not significantly affect black‐grass total, root or shoot biomass (Fig. 9d–f). Wheat biomass was not significantly inhibited by either biological or chemical treatment (Figure S3).

**Table 6:** Statistical outputs from ANOVA of mean black‐grass shoot, root, and total biomass after 3 weeks of growth under glasshouse conditions for both biological treatments (homogeneous black‐grass or mixed with wheat) and chemical treatments (no‐benzoxazinoid control, DIBOA or DIMBOA solution).

		sum sq	mean sq	No. df	denom df	*f*‐value	*P*‐value
shoots	Biological treatment	4.769e‐06	4.769e‐06	1	30	4.485	0.043*
	Chemical treatment	8.613e‐07	4.307e‐07	2	30	0.405	0.671
	Biological × chemical treatment	6.287e‐07	3.143e‐07	2	30	0.296	0.746
roots	Biological treatment	4.560e‐07	4.560e‐07	1	30	2.648	0.114
	Chemical treatment	1.504e‐08	7.520e‐09	2	30	0.044	0.957
	Biological × chemical treatment	9.836e‐08	4.918e‐08	2	30	0.2856	0.754
total	Biological treatment	8.175e‐06	8.175e‐06	1	30	4.9066	0.034*
	Chemical treatment	1.057e‐06	5.286e‐07	2	30	0.3172	0.731
	Biological × chemical treatment	2.745e‐07	1.372e‐07	2	30	0.0824	0.921

*P < 0.05.

## DISCUSSION

### Allelopathy of crude cereal root exudates to black‐grass

Crude cereal root exudates used here significantly and consistently inhibited black‐grass development. This corroborates previous reports of allelopathy from cereals against black‐grass and other weed species (Steinsiek *et al*. 1982; Bertholdsson 2012). Inhibition was mostly focused on roots, in accordance with general observations of comparative sensitivity to allelochemicals (Haugland & Brandsaeter 1996), although some significant effects on shoot length were also observed (e.g. Fig. 5a). This may be explained by the accumulation of high benzoxazinoid concentrations in root tissues following uptake (e.g. Hazrati *et al*. 2020; Hazrati *et al*. 2021). The inhibitory concentration of specific benzoxazinoids varies greatly according to target plant species, however (e.g. Macías *et al*. 2006), as noted for other allelochemicals (Nimbal *et al*. 1996).

### Diversity in root exudate benzoxazinoid content across wheat germplasm

A distinction among root exudation profiles between different wheat biotypes is suggested by our results. The apparent absence of DIMBOA‐related compounds in ‘MDR’‐line ancestor wheat root exudates distinguishes them from Watkins landraces and modern wheats, where MBOA, and DIMBOA‐Glc, were consistently putatively identified. MBOA is the direct degradation product of DIMBOA, and therefore indicative of its exudation (Macías *et al*. 2004), although alternative synthesis pathways are also suggested (Carlsen *et al*. 2009). *In planta* conversion of DIBOA to DIMBOA requires action of the genes *Bx6* and *Bx*7 (Jonczyk *et al*. 2008) (or their homologues). Therefore, it may be that their activity has evolved through domestication, given that compounds related to DIMBOA were only detected in hexaploid wheat.

Conversely, the apparent absence of DIBOA in root exudates of most modern commercial wheats corroborates previous reports on this compound as a relatively minor constituent of wheat, compared to DIMBOA (e.g. Pérez & Ormeño‐Nuñez 1991). There is a precedent for variation in root exudation profiles and concentrations between ancestor and modern crops, with consequences for defence capability (e.g. Maver *et al*. 2020). As hypothesised, root exudate composition of modern wheat cultivars appears to be relatively consistent, given genetic bottlenecks and use of common ancestors in breeding programmes (Haudry *et al*. 2007). On the other hand, these exudates seemed to contain the highest diversity of benzoxazinoid compounds, indicative of multiple pathways being bred into the modern wheat genome from multiple ancestors. Some variation in exudation profile among biotypes should be expected, however, as the benzoxazinoid exudation profile is dependent on past selection pressures. In an intriguing example, wild emmer wheat, *T. dicoccoides*, only synthesised DIMBOA when originating from locations where powdery mildew was a prevalent stressor (Ben‐Abu *et al*. 2018). Thus, the variant exudate profiles of wheat ancestors are unlikely to be fully understood without examination of their original habitats. Nonetheless, ancestral wheat germplasm has rarely been used for breeding in the modern era (Jing *et al*. 2007), so consideration of its exudation chemistry and allelopathic potential may benefit efforts to breed more effective weed‐suppressing lines.

Existing literature indicates that the effects of DIBOA‐Glc are comparable to those of DIBOA aglucone (Macías, Chinchilla, *et al*. 2005; Macías *et al*. 2006). Yenish *et al*. (1995) described in‐field weed suppression by rye residues containing DIBOA‐Glc, indicating its potency in the context of a biological treatment. The tentative identification of HBOA‐Glc in rye and ancestor wheat root exudates in our study is also notable, given their very effective inhibition of black‐grass; although, for both of these compounds, rapid degradation into aglucone forms indicates that the glucosides are not influential allelochemicals in surrounding plants (Macías *et al*. 2004; Macías, Oliveros‐Bastidas, *et al*. 2005). In this case, DIBOA and HBOA would be more long‐lived, but they (or their glucosides) might be synergised in crude root exudates to inhibit target species more effectively. Indeed, existing literature usually describes glucosides as storage compounds that prevent autotoxic effects of their constituent benzoxazinoids, with deglucosylation being a precursor to exudation (von Rad *et al*. 2002). More recently, however, these compounds have been found in *ex planta* root exudates (Hazrati *et al*. 2021; Mwendwa *et al*. 2021), suggesting a more complex, but currently unknown, role in chemical ecology.

While much has been published on the allelopathy of benzoxazinoids and other constituent compounds of crude cereal root exudates, like phenolic acids (e.g. Wu *et al*. 2001), there is debate concerning their relative importance as allelochemicals (Blum *et al*. 1999), as well as the potential contributions of other, currently unidentified, compounds. On this subject, Jia *et al*. (2006) examined the relative effects and potential for synergy between benzoxazinoid and phenolic allelochemicals, reporting that these compounds were likely to be additive or even antagonistic, but that benzoxazinoids play a larger role in the allelopathic interactions observed.

### Allelochemical stability in soil: An ongoing quandary

Many studies have described the swift degradation of benzoxazinoids in soil, and the degradation products produced (Macías *et al*. 2004; Macías, Oliveros‐Bastidas, *et al*. 2005). While essential to understanding the dynamics of these compounds, this speaks little to the consequences for target species, as there has been limited focus on the time‐frame required for an allelochemical to inhibit a plant. Allelopathy is more potent among young plants (Zhang *et al*. 2021), but myriad factors influence plant health and will also affect this interaction, highlighting the importance of examinations in biologically active systems.

Our results on the effect of biologically active soil on cereal allelopathy indicate that crude root exudates containing benzoxazinoid compounds can remain allelopathic (see Fig. 6), coherent with the potency of, e.g., rye root exudates for in‐field weed suppression (e.g. Tabaglio *et al*. 2008). On the other hand, our screening of individual benzoxazinoids in soils demonstrated that DIMBOA and DIBOA were ineffective towards black‐grass, in spite of their recorded effectiveness elsewhere (e.g. Yang *et al*. 2020). These data corroborate previous findings that benzoxazinoids are unstable in microbially‐active soils (e.g. Macías *et al*. 2004; Macías, Oliveros‐Bastidas, *et al*. 2005) or, alternatively, that other compounds in crude root exudates are contributors. Further work is also required to explore the influence of physicochemical factors, which differed between soil treatments and may have affected allelopathic potential. Kaur *et al*. (2009) estimated that only ca. 20% of allelopathic potential from axenic systems can be expected in biologically active soil media, and the dominant mitigating factor is likely microbial degradation (Cipollini *et al*. 2012), although sorption onto soil colloids can also play a role (Inderjit & Bhowmik 2004). Such research could employ an inoculum produced from such soils, which could then be delivered into a standard, sterilised medium to remove the influence of edaphic properties on allelochemical degradation (Robinson *et al*. 2021).

### Strength in diversity? Crude root exudates are more inhibitory than individual constituent compounds

Unlike the application of pure synthetic DIMBOA or DIBOA, wheat plants remained inhibitory to black‐grass development under glasshouse conditions, while the crop itself was not inhibited by any treatment. This experimental design did not attempt to remove resource competition, but inhibition of black‐grass shoot length by wheat 7 days after treatment had diminished at 14 days, which could indicate allelopathy. This is because allelochemical exudation is higher in younger plants (Copaja *et al*. 1999), and thus the target (black‐grass) is more likely to be sensitive to such chemicals at this life stage (Zhang *et al*. 2021). This differs from resource competition which, logically, should be more intense as plants grow larger and the growth space is reduced. When considering the presence of unidentified (likely non‐benzoxazinoid) compounds in allelopathic cereal root exudates, cereal allelopathy in soil is unlikely to be contingent on a single compound, but rather a diverse cocktail of chemicals that are exuded continuously (e.g. Reigosa *et al*. 1999; Jia *et al*. 2006). This indicates the value of natural chemodiversity in plant defence. Such natural chemodiversity also aligns with known allelochemical concentrations: the few studies quantifying exudation of specific benzoxazinoid compounds have reported concentrations below those at which inhibitory effects might be expected (Zhang *et al*. 2016; Hussain *et al*. 2022). The stronger effects exerted by crude root exudates may also be related to the slower degradation of allelochemicals because they contain more amenable target compounds for microbial metabolism, such as sugars, which could, incidentally, explain the effectiveness of root exudates containing glucosides found in this study.

## CONCLUSIONS

The results presented here clearly indicate that chemodiversity and allelopathic potential of crude cereal root exudates towards black‐grass vary across diverse germplasm, providing insight into how secondary metabolite defence profiles have altered during modern breeding. We also report, in line with previous results, resistance to other biotic stressors, where some ancestral wheat lines have greater allelopathic potential than intensively bred modern varieties. As such, we recommend that such germplasm should be considered for integration into breeding programmes to develop wheat lines with increased defence potential against weeds. Clearly, crude cereal root exudates are more effective plant inhibitors than individual, constituent inhibitory compounds. Thus, use of existing plant–plant interactions for weed management is preferable, using the natural chemodiversity of cereal crops (either actively growing or decomposing as a component of mulch), rather than applying individual constituent compounds.

## Author Contributions

DTH performed the study, including data analyses. MAB, AR, PN, KR, and DC supervised the study. JCC performed LCMS analyses; JCC and DMW assisted with isolation of root exudate samples. DMW synthesised chemical treatments. DTH wrote the initial draft of the manuscript. All authors contributed to the editing of the manuscript.

## Supporting information


**Figure S1.** Schematic of numbered plots from which soil was collected for Highfield soils assay, coloured by land use treatment.

**Figure S2.** LC‐MS Chromatograms of (a) autoclaved coarse sand, and (b) Weed mix soil media treated with DIMBOA, and (c) autoclaved coarse sand and (d) Highfield bare fallow soil media treated with DIBOA, across days 1–3 and day 7, all compared with benzoxazinoid standards.

**Figure S3.** Mean biomass of wheat after three weeks growth under glasshouse conditions under various treatments: (a) total biomass, (b) shoot biomass, and (c) root biomass by biological treatment; (d) total biomass, (e) shoot biomass and (f) root biomass by benzoxazinoid chemical treatment.

**Appendix S1.** Supplementary material.
