# Supplementary figures and images for: Variation in suppression of black‐grass by modern and ancestral cereal root exudates
Source: Plant Biol (Stuttg). 2025 Mar 26;27(5):802–17. doi: 10.1111/plb.70010 (PMC12255286; doi:10.1111/plb.70010)

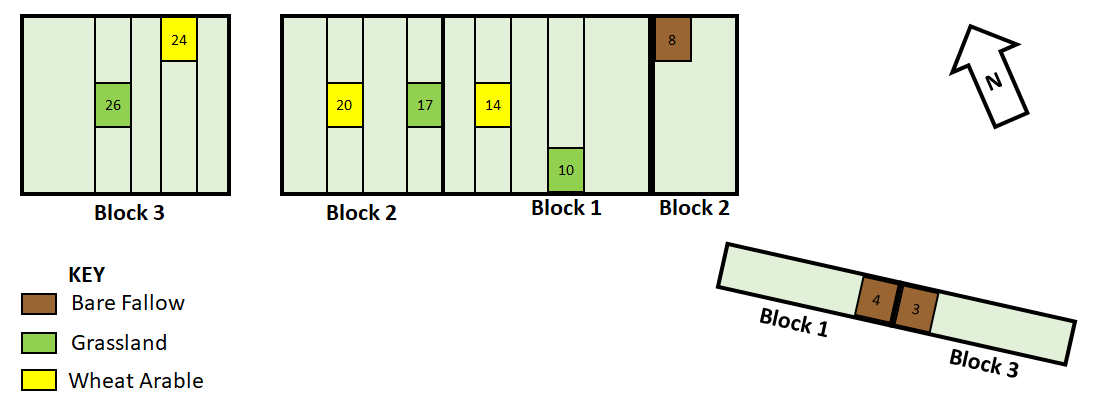

Supplement: Supplementary file 1 — Figure S1. Schematic of numbered plots from which soil was collected for Highfield soils assay, coloured by land use treatment. [file PLB-27-802-s004.png]

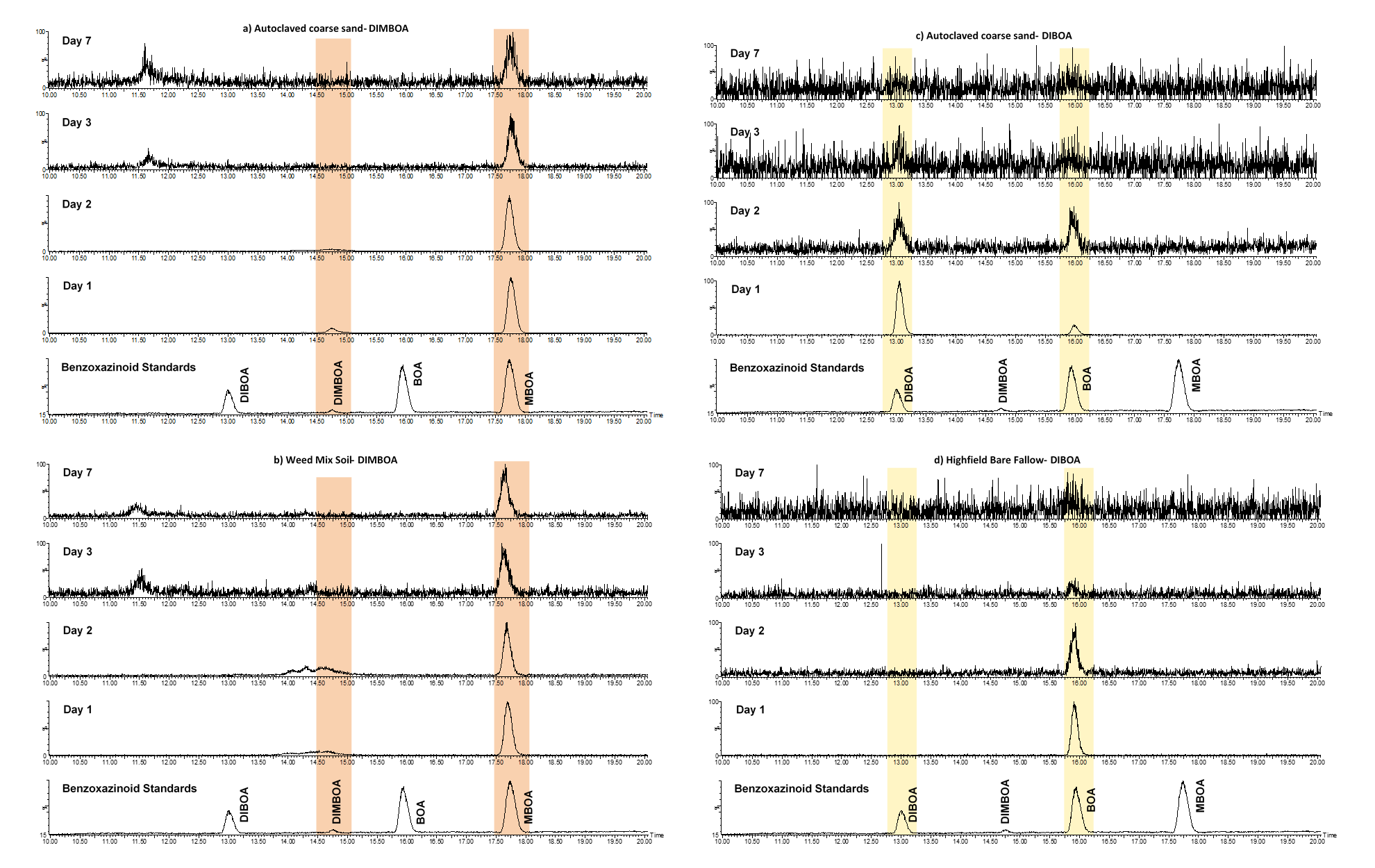

Supplement: Supplementary file 2 — Figure S2. LC‐MS Chromatograms of (a) autoclaved coarse sand, and (b) Weed mix soil media treated with DIMBOA, and (c) autoclaved coarse sand and (d) Highfield bare fallow soil media treated with DIBOA, across days 1–3 and day 7, all compared with benzoxazinoid standards. [file PLB-27-802-s001.png]

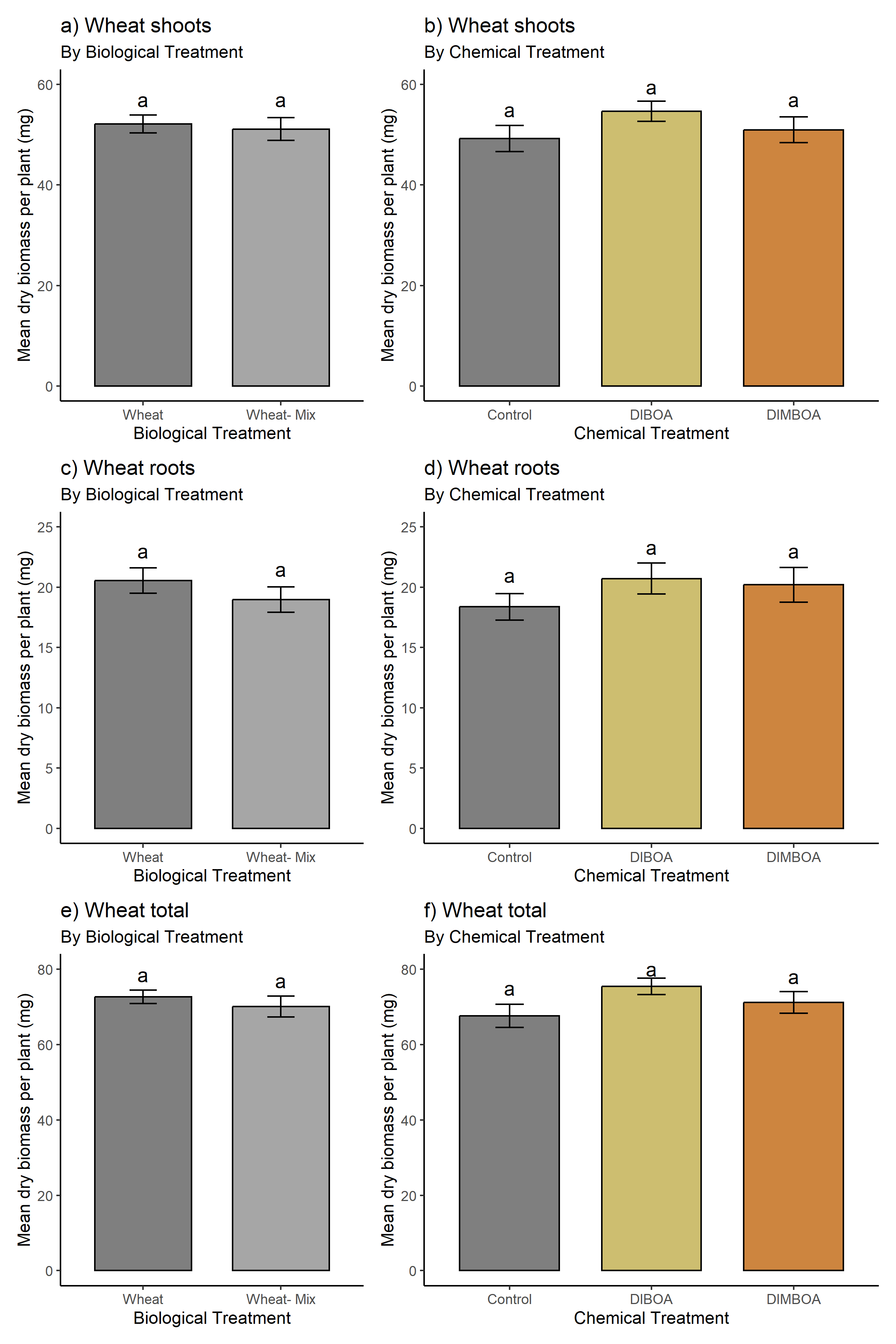

Supplement: Supplementary file 3 — Figure S3. Mean biomass of wheat after three weeks growth under glasshouse conditions under various treatments: (a) total biomass, (b) shoot biomass, and (c) root biomass by biological treatment; (d) total biomass, (e) shoot biomass and (f) root biomass by benzoxazinoid chemical treatment. [file PLB-27-802-s003.png]
