# Supplementary material for: Variation in suppression of black‐grass by modern and ancestral cereal root exudates
Source: Plant Biol (Stuttg). 2025 Mar 26;27(5):802–17. doi: 10.1111/plb.70010 (PMC12255286; doi:10.1111/plb.70010)
Supplement: Supplementary file 4 — Appendix S1. Supplementary material. [file PLB-27-802-s002.docx]

# Appendices

**Appendix S1: Methodological Tables and Figures**

Table S1: Summary table of wheat biotypes screened for variation in benzoxazinoid exudation profile.

| **Wheat Biotype** | **Classification** | **Experiment Screened in** |
| --- | --- | --- |
| **‘258’** | Watkins Landrace  (*T. aestivum*) | Ancestor Screen |
| **‘546’** |  |  |
| **‘624’** |  |  |
| **‘777’** |  |  |
| **‘821’** |  |  |
| **‘MDR031’** | Ancestor  (*T. monococcum*) |  |
| **‘MDR037’** |  | Both Screens (Ancestor Control) |
| **‘MDR043’** |  | Ancestor Screen |
| **‘MDR045’** |  |  |
| **‘MDR049’** |  |  |
| **‘Gravity’** | Modern Commercial  (*T. aestivum*) | Both Screens (Modern Control) |
| **‘Cadenza’** |  | Commercial Screen |
| **‘Costello’** |  |  |
| **‘Firefly’** |  |  |
| **‘Gleam’** |  |  |
| **‘Graham’** |  |  |
| **‘Siskin’** |  |  |
| **‘Skyscraper’** |  |  |
| **‘Spotlight’** |  |  |
| **‘Zyatt’** |  |  |

Table S2: Summary table of ‘Donors’ (pre-treatment cultures, grown for collection of crude root exudates), including where LC-MS analyses were undertaken, and ‘Recipient’ plant biotypes tested for sensitivity against each of these crude exudates.

| **Culture Name** | **‘Donor’ Contents, per tube** | **‘Donor’ Exudates Analysed?** | **Media used for bioassay** | **‘Recipient(s)’ Contents Screened,**  **per tube** |
| --- | --- | --- | --- | --- |
| **Control** | N/A | Y | Autoclaved coarse sand, ‘Weed mix’ agricultural soil | 4x *A. myosuroides* Rothamsted-17 seedlings, 4x *A. myosuroides* Peldon-13 seedlings, 4x *T. aestivum* var. Gravity seedlings |
| **Rothamsted**  **Black-grass** | 4x *A. myosuroides* Rothamsted-17 seedlings | Y |  |  |
| **Gravity wheat** | 4x *T. aestivum* var. Gravity seedlings | Y |  | 4x *A. myosuroides* Rothamsted-17 seedlings,4x *A. myosuroides* Peldon-13 seedlings |
| **Edmondo rye** | 4x *S. cereale* var. Edmondo seedlings | Y |  |  |
| **MDR037 wheat** | 4x *T. monococcum* var. *macedonicum* line MDR037 seedlings | Y |  | 4x *A. myosuroides* Rothamsted-17 seedlings |
| **MDR043 wheat** | 4x *T. monococcum* var. *monococcum* line MDR043 seedlings | Y | Autoclaved coarse sand |  |
| **MDR049 wheat** | 4x *T. monococcum* var. *monococcum* line MDR049 seedlings | Y |  |  |
| **Wheat/Black-grass mix** | 2x *T. aestivum* var. Gravity seedlings + 2x *A. myosuroides* Rothamsted-17 seedlings | Y |  | N/A  (Only grown for LC-MS characterisation of crude root exudates) |
| **Rye/Black-grass mix** | 2x *S. cereale* var. Edmondo seedlings +  2x *A. myosuroides* Rothamsted-17 seedlings | Y |  |  |

Figure S1: Schematic of numbered plots from which soil was collected for Highfield soils assay, coloured by land use treatment.

**Appendix S2: Supplementary Data and Results**

### S2.1: Variation in root exudate benzoxazinoid profile in wheat germplasm

Benzoxazinoid profiles showed limited variation between commercial wheat varieties (Table S3a); HBOA-Glc, DIBOA-Glc, HMBOA-Glc, BOA (2-benzoxazolinone) and MBOA, along with HDMBOA-Glc, were tentatively identified in all the nine varieties screened including Gravity, while DIMBOA-Glc was detected in all varieties excluding Gleam, Siskin and Gravity (Table S3a). In ancestral lines, similar benzoxazinoids were detected in *T. aestivum* and *T. monococcum* MDR037 as for previous analysis described above, apart from additional detection of DIBOA. *T. monococcum* MDR043, MDR045, and MDR049 contained similar benzoxazinoid profiles as *T. monococcum* MDR037. Benzoxazinoid profiles varied between Watkins collection landraces, but notably all landraces produced HMBOA-Glc and DIMBOA-Glc as for *T. aestivum* var. Gravity. There was no detection of DIBOA or DIMBOA aglucones.

Table S3: a) Presence/ absence of benzoxazinoid compounds in root exudates of wheat lines and cultivars used in commercial wheat screening, and b) of those used in screening of ancestral lines. Presence is indicated by the darker shade of grey, detection of trace quantities is indicated by the lighter shade of grey, and absence is indicated by a white box.

| **a)** | **Ancestors** | **Modern** | | | | | | | | |
| --- | --- | --- | --- | --- | --- | --- | --- | --- | --- | --- |
|  | **MDR037** | **Cadenza** | **Costello** | **Gleam** | **Graham** | **Gravity** | **Siskin** | **Skyscraper** | **Spotlight** | **Zyatt** |
| **MBOA** |  |  |  |  |  |  |  |  |  |  |
| **BOA** |  |  |  |  |  |  |  |  |  |  |
| **DIBOA** |  |  |  |  |  |  |  |  |  |  |
| **DIMBOA** |  |  |  |  |  |  |  |  |  |  |
| **DIBOA-Glc** |  |  |  |  |  |  |  |  |  |  |
| **HBOA-Glc** |  |  |  |  |  |  |  |  |  |  |
| **HMBOA-Glc** |  |  |  |  |  |  |  |  |  |  |
| **DIMBOA-Glc** |  |  |  |  |  |  |  |  |  |  |
| **HDMBOA-Glc** |  |  |  |  |  |  |  |  |  |  |

| **b)** | **Modern** | **Ancestors** | | | | | **Watkins Landraces** | | | | |
| --- | --- | --- | --- | --- | --- | --- | --- | --- | --- | --- | --- |
|  | **Gravity** | **MDR031** | **MDR037** | **MDR043** | **MDR045** | **MDR049** | **258** | **546** | **624** | **777** | **821** |
| **MBOA** |  |  |  |  |  |  |  |  |  |  |  |
| **BOA** |  |  |  |  |  |  |  |  |  |  |  |
| **DIBOA** |  |  |  |  |  |  |  |  |  |  |  |
| **DIMBOA** |  |  |  |  |  |  |  |  |  |  |  |
| **DIBOA-Glc** |  |  |  |  |  |  |  |  |  |  |  |
| **HBOA-Glc** |  |  |  |  |  |  |  |  |  |  |  |
| **HMBOA-Glc** |  |  |  |  |  |  |  |  |  |  |  |
| **DIMBOA-Glc** |  |  |  |  |  |  |  |  |  |  |  |

### S2.2: Benzoxazinoid degradation in soil: Methodology and results

To accompany experiment 5 (benzoxazinoid testing in soils), DIMBOA and DIBOA degradation was tracked in the four soils described above along with autoclaved coarse sand as a negative control. Eppendorf tubes (2 ml) were filled with either soil (0.5 g per tube) sand, wetted to 25% of WHC, and incubated in the axenic tube assay set up for 14 days, upon which either DIBOA or DIMBOA were added. Three replicates were tested for each treatment combination (*n*= 30). Tubes were then sealed and incubated for a further seven days. On days 1, 2, 3, and 7, soils and sand were eluted with LC-MS-grade methanol (0.5g, 99%). Eluates were vortexed for 30 seconds, then centrifuged for 15 minutes at 3,000 rpm, and pipetted for LC-MS analysis. Analysis and detection by LC-MS were undertaken as described in the main text.

Examination of DIMBOA and DIBOA degradation in the four tested soils, and the additional negative control of autoclaved coarse sand, identified correlations between allelopathic potential of these compounds and degradation rate. DIMBOA degradation was largely consistent in the five media (Figure S2a and S2b); in autoclaved coarse sand, DIMBOA was still identified at Day 3, but in all other media, its peak had diminished by this time. MBOA was identified at all time points in all media where DIMBOA had been applied. The degradation rate of DIBOA varied more greatly between media; it was still recovered after three days in autoclaved coarse sand (Figure S2c), while its degradation product BOA was also identified on all days. All Highfield soils degraded DIBOA to BOA entirely within two days, and within one day in bare fallow soil (Figure S2d).

Figure S2: LC-MS Chromatograms of a) autoclaved coarse sand, and b) Weed mix soil media treated with DIMBOA, and c) autoclaved coarse sand and d) Highfield bare fallow soil media treated with DIBOA, across days 1 to 3 and day 7, all compared with benzoxazinoid standards. Sensitivity was increased as days advanced to facilitate the identification of corresponding peaks; sensitivity values are provided on the top left of each graph*.* Methodology and results provided below figure.

### S2.3: Wheat biomass from glasshouse assays: Results

Figure S3: Mean biomass of wheat after three weeks growth under glasshouse conditions under various treatments: a) total biomass, b) shoot biomass, and c) root biomass by biological treatment; d) total biomass, e) shoot biomass and f) root biomass by benzoxazinoid chemical treatment. Pot replicates= 6 (*n* = 54), Individual seedlings per pot= 6 (or 3+3 where mixed). Error bars indicate SEMs, lettering indicates that no significant differences were found from corresponding controls.

Wheat biomass was not significantly inhibited by biological treatment (Figures S3a to S3c), or chemical treatment (Figures S3d to S3f; see also Table S4). There was also no significant interaction between these two factors in any metric, so chemical treatments did not differentially affect wheat in isolation compared to in the presence of black-grass.

Table S4: Statistical outputs from ANOVA analyses of mean wheat shoot, root, and total biomass after three weeks growth under glasshouse conditions under both biological treatments (homogeneous wheat or mixed with black-grass), and chemical treatments (no-benzoxazinoid control, DIBOA or DIMBOA solution).

|  |  | Sum Sq | Mean Sq | Num Df | Denom Df | *f*-value | *p*-value |
| --- | --- | --- | --- | --- | --- | --- | --- |
| Shoots | Biological Treatment | 9.201e-06 | 9.201e-06 | 1 | 25 | 0.145 | 0.707 |
|  | Chemical Treatment | 1.861e-04 | 9.307e-05 | 2 | 25 | 1.463 | 0.251 |
|  | Biological x Chemical Treatment | 1.299e-04 | 6.499e-05 | 2 | 25 | 1.021 | 0.375 |
| Roots | Biological Treatment | 2.302e-05 | 2.3024e-05 | 1 | 25 | 1.737 | 0.441 |
|  | Chemical Treatment | 3.606e-05 | 1.8028e-05 | 2 | 25 | 1.361 | 0.183 |
|  | Biological x Chemical Treatment | 8.580e-06 | 4.2898e-06 | 2 | 25 | 0.324 | 0.470 |
| Total | Biological Treatment | 0.0000613 | 6.1335e-05 | 1 | 25 | 0.613 | 0.441 |
|  | Chemical Treatment | 0.0003646 | 1.8231e-04 | 2 | 25 | 1.822 | 0.183 |
|  | Biological x Chemical Treatment | 0.0001565 | 7.8265e-05 | 2 | 25 | 0.782 | 0.468 |

## Appendix S3: Synthesis of DIMBOA and DIBOA

### S3.1. Synthesis of potassium 2-nitrophenolate

To a solution of 2-nitrophenol (5 g, 35.94 mmol) in DCM (15 ml) was added potassium hydroxide (2.02 g, 35.94 mmol) in water (10 ml) and the solution vigorously stirred for 16 hours. The solvent was removed under vacuum before drying further in a vacuum desiccator for 3 days to give potassium 2-nitrophenolate (6.32 g, 99% yield) as an orange solid.

**^1^H-NMR (d_6_-DMSO, 500 MHz):** 7.66 (d, 1H, *J = 8.4 Hz*), 7.04 (t, 1H, *J = 7.5 Hz*), 6.52 (d, 1H, *J = 8.7 Hz*), 6.08 (t, 1H, *J = 7.5 Hz*).**^13^C-NMR (d_6_-DMSO, 125 MHz):** 166.54, 136.70, 134.22, 126.81, 126.73, 109.55

### S3.2. Synthesis of methyl 2-methoxy-2-(2-nitrophenoxy)acetate

To a solution of methylmethoxyacetate (4.56 g, 43.86 mmol) and N-bromosuccinimide (7.81 g, 43.86 mmol) in carbon tetrachloride (30 ml) was added dibenzoyl peroxide (104 mg, 0.43 mmol) and the mixture heated to 80 ^o^C for 2 hours. The reaction mixture was cooled to 0 ^o^C for 15 mins before being filtered through cotton wool directly into a suspension of potassium 2-nitrophenolate (5 g, 29.24 mmol) in THF (60 ml) and stirred for a further 16 hours. The reaction mixture was diluted with DCM before being washed with water, dried (MgSO_4_) and concentrated under vacuum. The crude product was purified on silica gel (25% EtOAc in pet ether) to give methyl 2-methoxy-2-(2-nitrophenoxy)acetate (5.56 g, 79% yield) as a pale yellow solid.

**^1^H-NMR (CDCl_3_, 500 MHz):** 7.87 (dd, 1H, *J = 8.2, 1.7 Hz*), 7.55 (td, 1H, *J = 8.1, 1.8 Hz*), 7.32 (dd, 1H, *J = 8.5, 0.9 Hz*), 7.20 (td, 1H, *J = 8.5, 0.9 Hz*), 5.60 (s, 1H), 3.86 (s, 3H), 3.62 (s, 3H). **^13^C-NMR (CDCl_3_, 125 MHz):** 165.91, 148.81, 133.93, 125.52, 123.09, 118.97, 98.64, 55.19, 53.04

### S3.3. Synthesis of 4-hydroxy-2-methoxy-2H-benzo[1,4]oxazin-3(4H)-one

To a vigorously stirred suspension of 10% Pd/C (200 mg) in 1:1 1,4-dioxane:water (180 ml) was added sodium borohydride (1.05 g, 27.67 mmol). Methyl 2-methoxy-2-(2-nitrohenoxy)acetate (5.56 g, 23.06 mmol) in 1,4-dioxane (10 ml) was added dropwise and the reaction mixture stirred for a further 30 mins after complete addition. The reaction mixture was filtered through celite and the filtrate pH adjusted to 3 with 2M HCl before being extracted with EtOAc. The combined organics were dried (MgSO_4_) and concentrated under vacuum. The crude product was purified on silica gel (35% EtOAc in pet ether) to give 4-hydroxy-2-methoxy-2H-benzo[1,4]oxazin-3(4H)-one (3.22 g, 72% yield) as a pink solid.

**^1^H-NMR (d_6_-DMSO, 500 MHz):** 11.07 (bs, 1H), 7.29 (m, 1H), 7.14 (m, 2H), 7.09 (m, 1H), 5.57 (s, 1H), 3.45 (s, 3H).**^13^C-NMR (d_6_-DMSO, 125 MHz):** 156.31, 140.42, 129.19, 124.60, 123.64, 117.64, 113.61, 98.25, 56.32.

### S3.4. Synthesis of 2,4-dihydroxy-2H-benzo[1,4]oxazin-3(4H)-one (DIBOA)

To a solution of 4-hydroxy-2-methoxy-2H-benzo[d][1,4]oxazin-3(4H)-one (3 g, 15.38 mmol) in DCM (100 ml), cooled to -50 ^o^C under N_2_, was added precooled 1M boron trichloride solution in DCM (46.1 ml, 46.14 mmol). The reaction mixture was allowed to warm to RT over 3 hours before THF (30 ml) was added, poured into water and extracted with EtOAc. The combined organic layers were concentrated to ~20 ml under vacuum and diluted with THF (20 ml). This solution was added to a vigorously stirred suspension of silver carbonate (8.48 g, 30.76 mmol) in 2:1 water:THF (20 ml) and stirred for 30 mins. The mixture was filtered and extracted with EtOAc. The combined organics were dried (MgSO_4_) and concentrated to ~25 ml under vacuum. Hexane was added dropwise to initiate crystallisation, after which the mixture was placed in a freezer for 3 days. Collection of the precipitate gave DIBOA (2.10 g, 76% yield) as an off white solid.

### S3.5. Synthesis of potassium 5-methoxy-2-nitrophenolate

To a solution of 5-methoxy-2-nitrophenol (5 g, 29.56 mmol) in DCM (15 ml) was added potassium hydroxide (1.66 g, 29.56 mmol) in water (10 ml) and the solution vigorously stirred for 16 hours. The solvent was removed under vacuum before drying further in a vacuum desiccator for 3 days to give potassium 5-methoxy-2-nitrophenolate (5.91 g, 97% yield) as an orange solid.

**^1^H-NMR (d_6_-DMSO, 500 MHz):** 7.63 (d, 1H, *J = 9.5* Hz), 5.78 (d, 1H, *J = 2.3* Hz), 5.61 (dd, 1H, *J = 9.5, 2.3* Hz). **^13^C-NMR (d_6_-DMSO, 125 MHz):** 170.57, 164.55, 130.93, 128.36, 106.35, 101.19, 55.06

### S3.6. Synthesis of methyl 2-methoxy-2-(5-methoxy-2-nitrophenoxy)acetate

To a solution of methylmethoxyacetate (1 g, 9.60 mmol) and N-bromosuccinimide (1.71 g, 9.60 mmol) in carbon tetrachloride (20 ml) was added dibenzoyl peroxide (20 mg, 0.08 mmol) and the mixture heated to 80 ^o^C for 2 hours. The reaction mixture was cooled to 0 ^o^C for 15 mins before being filtered through cotton wool directly into a suspension of potassium 5-methoxy-2-nitrophenolate (2.19 g, 10.56 mmol) in THF (12 ml) and stirred for a further 2 hours. The reaction mixture was diluted with DCM before being washed with water, dried (MgSO_4_) and concentrated under vacuum. The crude product was purified on silica gel (25% EtOAc in pet ether) to give methyl 2-methoxy-2-(5-methoxy-2-nitrophenoxy)acetate (1.89 g, 73% yield) as a pale yellow solid.

**^1^H-NMR (CDCl_3_, 500 MHz):** 7.99 (d, 1H, *J = 9.3 Hz*), 6.78 (d, 1H, *J = 2.5 Hz*), 6.67 (dd, 1H, *J = 9.1, 2.5 Hz*), 5.59 (s, 1H), 3.88 (s, 3H), 3.86 (s, 3H), 3.62 (s, 3H).**^13^C-NMR (CDCl_3_, 125 MHz):** 165.94, 164.24, 151.41, 134.29, 128.06, 107.96, 104.73, 98.72, 56.03, 55.18, 53.03

### S3.7. Synthesis of 4-hydroxy-2,7-dimethoxy-2H-benzo[1,4]oxazin-3(4H)-one

To a vigorously stirred suspension of 10% Pd/C (32 mg) in 1:1 1,4-dioxane:water (32 ml) was added sodium borohydride (167 g, 4.42 mmol). Methyl 2-methoxy-2-(5-methoxy-2-nitrophenoxy)acetate (1 g, 3.68 mmol) in 1,4-dioxane (3 ml) was added dropwise and the reaction mixture stirred for a further 30 mins after complete addition. The reaction mixture was filtered through celite and the filtrate pH adjusted to 3 with 2M HCl before being extracted with EtOAc. The combined organics were dried (MgSO_4_) and concentrated under vacuum. The crude product was purified on silica gel (40% EtOAc in pet ether) to give 4-hydroxy-2,7-dimethoxy-2H-benzo[1,4]oxazin-3(4H)-one (501 mg, 61% yield) as a pink solid.

### S3.8. Synthesis of 2,4-dihydroxy-7-methoxy-2H-benzo[1,4]oxazin-3(4H)-one (DIMBOA)

To a solution of 4-hydroxy-2,7-dimethoxy-2H-benzo[1,4]oxazin-3(4H)-one (501 mg, 2.23 mmol) in DCM (20 ml), cooled to -50 ^o^C under N_2_, was added precooled 1M boron trichloride solution in DCM (6.7 ml, 6.69 mmol). The reaction mixture was allowed to warm to RT over 3 hours before THF (30 ml) was added, poured into water and extracted with EtOAc. The combined organic layers were concentrated to ~5 ml under vacuum and diluted with THF (4 ml). This solution was added to a vigorously stirred suspension of silver carbonate (1.23 g, 4.46 mmol) in 2:1 water:THF (6 ml) and stirred for 30 mins. The mixture was filtered and extracted with EtOAc. The combined organics were dried (MgSO_4_) and concentrated to ~2 ml under vacuum. Hexane was added dropwise to initiate crystallisation, after which the mixture was placed in a freezer for 3 days. Collection of the precipitate gave DIBOA (189 mg, 40% yield) as a brown solid.
